# Treatment and surveillance for non-muscle-invasive bladder cancer: a clinical practice guideline (2021 edition)

**DOI:** 10.1186/s40779-022-00406-y

**Published:** 2022-08-17

**Authors:** Ying-Hui Jin, Xian-Tao Zeng, Tong-Zu Liu, Zhi-Ming Bai, Zhong-Ling Dou, De-Gang Ding, Zhi-Lu Fan, Ping Han, Yi-Ran Huang, Xing Huang, Ming Li, Xiao-Dong Li, Yi-Ning Li, Xu-Hui Li, Chao-Zhao Liang, Jiu-Min Liu, Hong-Shun Ma, Juan Qi, Jia-Qi Shi, Jian Wang, De-Lin Wang, Zhi-Ping Wang, Yun-Yun Wang, Yong-Bo Wang, Qiang Wei, Hai-Bo Xia, Jin-Chun Xing, Si-Yu Yan, Xue-Pei Zhang, Guo-You Zheng, Nian-Zeng Xing, Da-Lin He, Xing-Huan Wang, the Chinese Urological Doctor Association (CUDA), Urological Association of Chinese Research Hospital Association (CRHA-UA), Uro-Health Promotive Association of China International Exchange, Promotive Association for Medical, Health Care (CPAM-UHPA)

**Affiliations:** 1 Center for Evidence-Based and Translational Medicine, Zhongnan Hospital of Wuhan University, Wuhan, 430071 China; 2 Department of Urology, Institute of Urology, Zhongnan Hospital of Wuhan University, Wuhan, 430071 China; 3 Department of Urology, Haikou People’s Hospital, Haikou Affiliated Hospital of Central South University Xiangya School of Medicine, Haikou, 570208 China; 4 Department of Urology, the First Affiliated Hospital and College of Clinical Medicine, Henan University of Science and Technology, Luoyang, 450052 Henan China; 5 Department of Urology, Henan Provincial People’s Hospital, Zhengzhou University People’s Hospital, Zhengzhou, 450003 China; 6 Department of Urology, the Second Hospital of Dalian Medical University, Dalian, 116023 Liaoning China; 7 Department of Urology, Institute of Urology, West China Hospital, Sichuan University, Chengdu, 610041 China; 8 Department of Urology, Renji Hospital, School of Medicine, Shanghai Jiao Tong University, Shanghai, 200120 China; 9 Department of Urology, the People’s Hospital of Xinjiang Uygur Autonomous Region, Urumqi, 830002 China; 10 Department of Urology, Huaihe Hospital of Henan University, Kaifeng, 475000 Henan China; 11 Institutes of Evidence-Based Medicine and Knowledge Translation, Henan University, Kaifeng, 475000 Henan China; 12 Department of Urology, the Second Affiliated Hospital and Second Clinical Medical College of Fujian Medical University, Quanzhou, 362000 Fujian China; 13 Department of Urology, the First Affiliated Hospital of Anhui Medical University, Hefei, 230022 China; 14 Department of Urology, Guangdong Provincial People’s Hospital, Guangdong Academy of Medical Sciences, Guangzhou, 510080 China; 15 Department of Urology, Tianjin First Central Hospital, Tianjin, 300190 China; 16 Department of Urology, Xinhua Hospital, School of Medicine, Shanghai Jiao Tong University School of Medicine, Shanghai, 200092 China; 17 Department of Urology, Affiliated Hospital of Guizhou Medical University, Guiyang, 550004 China; 18 Department of Urology, Qinghai University Affiliated Hospital, Xining, 810012 China; 19 Department of Urology, the First Affiliated Hospital of Chongqing Medical University, Chongqing, 400042 China; 20 Department of Urology, Institute of Urology, Lanzhou University Second Hospital, Key Laboratory of Urological Diseases in Gansu Province, Lanzhou, 730030 China; 21 Department of Urology, Chifeng Cancer Hospital, the Second Affiliated Hospital of Chifeng University, Chifeng, 024000 Inner Mongolia Autonomous Region China; 22 Key Laboratory of Urinary Tract Tumors and Calculi, Department of Urology, the First Affiliated Hospital of Xiamen University, Xiamen, 361003 China; 23 Department of Urology, the First Affiliated Hospital of Zhengzhou University, Zhengzhou, 450052 China; 24 Department of Urology, the Second Division of the First Hospital of Jilin University, Changchun, 130061 China; 25 Department of Urology, National Cancer Center/National Clinical Research Center for Cancer/Cancer Hospital, Chinese Academy of Medical Sciences and Peking Union Medical College, Beijing, 100021 China; 26 Department of Urology, the First Affiliated Hospital of Xi’an Jiaotong University, Xi’an 710061, China

**Keywords:** Non-muscle invasive bladder cancer, Bladder cancer, Transurethral resection of bladder tumor, Treatment, Surveillance, Guideline

## Abstract

Non-muscle invasive bladder cancer (NMIBC) is a major type of bladder cancer with a high incidence worldwide, resulting in a great disease burden. Treatment and surveillance are the most important part of NIMBC management. In 2018, we issued “Treatment and surveillance for non-muscle-invasive bladder cancer in China: an evidence-based clinical practice guideline”. Since then, various studies on the treatment and surveillance of NMIBC have been published. There is a need to incorporate these materials and also to take into account the relatively limited medical resources in primary medical institutions in China. Developing a version of guideline which takes these two issues into account to promote the management of NMIBC is therefore indicated. We formed a working group of clinical experts and methodologists. Through questionnaire investigation of clinicians including primary medical institutions, 24 clinically concerned issues, involving transurethral resection of bladder tumor (TURBT), intravesical chemotherapy and intravesical immunotherapy of NMIBC, and follow-up and surveillance of the NMIBC patients, were determined for this guideline. Researches and recommendations on the management of NMIBC in databases, guideline development professional societies and monographs were referred to, and the European Association of Urology was used to assess the certainty of generated recommendations. Finally, we issued 29 statements, among which 22 were strong recommendations, and 7 were weak recommendations. These recommendations cover the topics of TURBT, postoperative chemotherapy after TURBT, Bacillus Calmette–Guérin (BCG) immunotherapy after TURBT, combination treatment of BCG and chemotherapy after TURBT, treatment of carcinoma in situ, radical cystectomy, treatment of NMIBC recurrence, and follow-up and surveillance. We hope these recommendations can help promote the treatment and surveillance of NMIBC in China, especially for the primary medical institutions.

**Supplementary Information:**

The online version contains supplementary material available at 10.1186/s40779-022-00406-y.

## Background

Bladder cancer is one of the most commonly diagnosed cancers worldwide, with more than 500 thousand newly diagnosed cases and 200 thousand deaths estimated globally in 2019 [1–3]. As one of the types of bladder cancer, non-muscle invasive bladder cancer (NMIBC) is the most common form of bladder cancer, comprising approximately 75% of cases [4]. NMIBC is characterized by frequent recurrences and a high risk of disease progression, as reported the 5-year recurrence rates ranging from 50 to 70%, and 5-year progression rates ranging from 10 to 30% [4, 5]. Proper management can reduce the risk of recurrence and progression of the disease. The management of NMIBC includes transurethral resection of bladder tumor (TURBT), intravesical chemotherapy and intravesical immunotherapy, and follow-up and surveillance of NMIBC patients [4]. In China, as the medical resources are distributed unbalancedly, there is a need to strengthen the management of NMIBC. For example, in some areas, there is a shortage of the Bacillus Calmette–Guérin (BCG) for NMIBC immunotherapy in the primary medical institutions. Therefore, it is necessary to develop a version of guidelines for different grade hospitals, especially including primary medical institutions to promote the management of NMIBC which takes into account the conditions prevailing there.

In 2018, we published a guideline for the treatment and surveillance of NMIBC in China [6]. Considering the studies on the treatment and surveillance of NMIBC being published in recent years, and the need to promote the management of NMIBC including primary medical institutions, we updated and developed 2021 edition of the guidelines for the treatment and surveillance of NMIBC in China. This guideline includes eight sections: TURBT, postoperative chemotherapy after TURBT, BCG immunotherapy after TURBT, combination treatment of BCG and chemotherapy after TURBT, treatment of carcinoma in situ (CIS), radical cystectomy, treatment of NMIBC recurrence, follow-up and surveillance.

## Methods

### Target users

The main users of the guideline are urologists, nursing staff, and general practitioners in different grade medical institutions that can provide NIMBC diagnosis and treatment services, especially at or below the county level. Other users include teachers and researchers working in the area of bladder cancer treatment.

### Target population

Patients with NMIBC.

### Composition of the guideline development group

Experts who were members of Project Groups for Minimally Invasive Plasma Surgery System of National Key Research and Development Program and Cloud Planning Solution, Professional Committee members of the Chinese Urological Doctor Association (CUDA), Urological Association of Chinese Research Hospital Association (CRHA-UA), Uro-Health Promotive Association of China International Exchange and Promotive Association for Medical and Health Care (CPAM-UHPA), and Evidence-based Medicine Branch of China International Exchange and the Promotive Association for Medical and Health Care composed the guideline steering committee, guideline development group and the guideline external review expert group.

The guideline panel was composed of a steering group, a working group, and an evidence search and synthesis group, which included 27 urological experts, 2 methodologists, and 16 clinical research assistants with evidence searching and assessment expertise. The external consultancy review group included 11 clinical experts and one methodologist. (See the Authors’ Contributions).

### Selection and identification of clinical questions and outcomes

The guideline working group designed a questionnaire for the selection and identification of clinical concerns and outcomes for the development of guideline through systematic retrieval of published guidelines and systematic reviews, stakeholder questionnaires, and conference discussions. It included 35 clinical questions on 8 topics: TURBT, postoperative chemotherapy after TURBT, BCG immunotherapy after TURBT, combination treatment of BCG and chemotherapy after TURBT, treatment of CIS, radical cystectomy, treatment of NMIBC recurrence, follow-up and surveillance. Each topic consists of two parts: one was to investigate the status of the topic-related concerns in clinical practice; the other was to evaluate the feasibility and importance of the topic-related clinical questions. A Likert 5-level scoring method was used to assess the importance of clinical questions, with 1 representing very unimportant and 5 representing very important.

From November 2020 to January 2021, a questionnaire survey was conducted using the designed questionnaires among 112 urologists in the county-level medical institutions, and the survey results were reported to the guideline development committee. After discussion, 11 clinical questions with an average score of less than 4, less application in clinical practice or duplication of contents were deleted, and a total of 24 clinical questions were included in the guidelines.

The outcome indicators in the guidelines were: (1) key outcome indicators: progression-free survival (PFS), overall survival (OS), recurrence-free survival (RFS) and cancer-specific survival (CSS). (2) Important outcome indicators: recurrence rate, total mortality and disease-specific mortality. (3) Adverse reactions: 1) adverse reactions related to adjuvant therapies, including: frequent urination, urodynia, pyuria, hematuria, dysuria, bladder irritation symptoms, cystitis, fever, allergic reaction, gastrointestinal reaction and general discomfort; 2) adverse reactions related to surgery operations: obturator nerve reflex, bladder perforation, urinary extravasation, bladder irritation symptoms, urinary tract infection and urinary tract stenosis.

### Evidence reviews

Evidence was searched from multiple resources, including: Databases (PubMed, Embase, Web of Science, The Cochrane Library, China National Knowledge Infrastructure, China Science and Technology Journal Database, Wanfang Data, Chinese BioMedical Literature Database), representative guideline development professional societies [European Association of Urology (EAU), American Urological Association (AUA), Canadian Urological Association (CUA), National Institute for Health and Clinical Excellence (NICE), Scottish Intercollegiate Guidelines Network (SIGN), National Comprehensive Cancer Network (NCCN)], monographs (Chinese Guidelines for Diagnosis and Treatment of Urology and Andrology [7], Standardization of Cancer Diagnosis and Treatment Series—bladder cancer and prostate cancer [8], Chinese Expert Consensus on Secondary Resection of Non-muscle-invasive Bladder Cancer [9]).

Systematic review and meta-analysis published in professional medical journals were first considered for evidence synthesis. If there was no relevant systematic review or meta-analysis on the topic, we would consider formulating one based on the existing primary research. If there was no relevant primary research, we would look for the published guidelines, consensus, monographs and expert opinions. The quality of evidence of primary research was evaluated according to the relevant criteria [10].

### Formation of recommendations

The grade criteria for evidence and recommendation used in the EAU guideline [11] (Table 1) were chosen for the formation of recommendations. Strong recommendations mean that most informed patients should be given the recommended management and that clinicians can organize their interactions with patients accordingly. Weak recommendations mean that the management given to the patients will vary depending on their values and preferences, and clinicians must ensure that patient’s care is in line with their values and preferences. We used the word “recommend” to introduce “strong recommendations”, and used “suggest” or “consider” to describe “weak recommendations”.

**Table 1:** Levels of evidence and grades of the recommendation in EAU Guideline

Level	Type of evidence
1a	Evidence obtained from meta-analysis of randomized trials
1b	Evidence obtained from at least one randomized trial
2a	Evidence obtained from one well-designed controlled study without randomization
2b	Evidence obtained from at least one other type of well-designed quasi-experimental study
3	Evidence obtained from well-designed non-experimental studies, such as comparative studies, correlation studies and case reports
4	Evidence obtained from expert committee reports or opinions or clinical experience of respected authorities

Grade	Nature of recommendation
Strong recommendation (for/against)	The advantages of interventions obviously outweigh the disadvantages or the disadvantages obviously outweigh the advantages
Weak recommendation (for/against)	The advantages and disadvantages of interventions are uncertain or the evidence regardless of its quality shows that the advantages and disadvantages are equal

*EAU* European Association of Urology

Consensus principles for recommendation voting were as follows: if the number of votes for the strength of a recommendation was more than 50% of total voters, the direction (such as support or oppose an intervention) and strength of recommendations can be determined directly; if the above standards cannot be met, but the total number of votes in the same direction of recommendation exceeds 70%, the direction of the recommendations can be determined, the strength of recommendations depends on the highest number of votes; if the above two items cannot be met, the next stage of discussion shall be performed to reach an agreement.

### Classification criterion of NMIBC

The staging of NMIBC in this guideline was based on Tumor Node Metastasis (TNM) staging method (Additional file 1: Table S1) of the Union for International Cancer Control (UICC)[12]. The histological grading of NMIBC was based on the WHO standard (2004/2016 version) (Additional file 1: Table S2) [13], but WHO/International Society of Urology standard (1973 version) (Additional file 1: Table S2) [14] was not excluded in the process of literature collection. The risk classification criterion for NMIBC in this guideline is shown in Additional file 1: Table S3. Additional file 1: Table S4 shows the risk classification criterion of EAU guideline [11].

## Results

### Section 1: Clinical concerns related to the surgical treatment of NMIBC

#### Question 1: What are the indications for TURBT in NMIBC patients?

Recommendation: For patients with suspected NMIBC, TURBT is recommended as the diagnosis procedure and initial treatment measure. (Evidence level: 4; Strength of recommendation: Strong).

Implementation consideration: If the conditions permit, remove all visible tumors through TURBT, and conduct a histological examination for pathological staging and grading.

Evidence summary: We referred to the recommendations from the EAU guideline [11], AUA guideline [15], CUA guideline [16], and Chinese Guidelines for Diagnosis and Treatment of Urology and Andrology [7] (Additional file 2: Question 1).

#### Question 2: What are the indications not to undergo TURBT as the primary procedure for patients with NMIBC?

Recommendation: TURBT is not recommended for patients with insurmountable issues that hinder the implementation of TURBT and for patients who require radical cystectomy. (Evidence level: 4; Strength of recommendation: Strong).

Implementation consideration: Insurmountable conditions hindering the implementation of TURBT include severe urinary tract stenosis, patients who cannot be placed in the lithotomy position due to skeletal or muscle disease. See Question 22 for indications for radical cystectomy.

Evidence summary: We referred to the recommendations from the Standardization of Cancer Diagnosis and Treatment Series—bladder cancer and prostate cancer [8]: Patients with severe urethral stricture, bladder adenocarcinoma, squamous cell carcinoma, bladder diverticulum cancer and urachal cancer, or patients who cannot be placed in the lithotomy position due to bone or muscle diseases, or those who relapse rapidly after the first treatment cannot undergo TURBT.

#### Question 3: What is the extent of initial TURBT resection in NMIBC patients?

Recommendation: Endoscopically visible tumors should be resected deep into underlying detrusor muscle in the initial TURBT resection. Tumors with a diameter less than 1 cm could be resected along with part of the bladder wall under the tumor. For large tumors, it is recommended to resect the tumor in fractions until normal bladder wall muscle is exposed. The biopsy specimens sent for pathological examination should include the muscular tissue. (Evidence level: 4; Strength of recommendation: Strong).

Evidence summary: We referred to the recommendations from the EAU guideline [11], CUA guideline [16], NICE guideline [17], NCCN guideline [18], and Guidelines for Diagnosis and Treatment of Urology and Andrology in China [7] (Additional file 2: Question 3).

#### Question 4: What are the indications for fluorescence- or narrow-band imaging-guided TURBT in patients with NMIBC?

Recommendation: If the equipment and operators are available, TURBT guided by fluorescence or narrow-band imaging can be used for patients suspected of having multiple tumors, CIS or high-grade tumors, or for patients with positive urine cytology but negative ordinary cystoscopy. (Evidence level: 1a; Strength of recommendation: Weak).

Implementation consideration: The commonly used photosensitizers in clinical practice during fluorescence guidance are 5-aminolevulinic acid (5-ALA) and hexaminolevulinate (HAL).

Evidence summary: We referred to the recommendations from relevant systematic reviews, guidelines and monographs. (1) A systematic review [19] published in 2021 recruited 20 RCTs (*n* = 5217), including 5-ALA vs. white light, HAL vs. white light and narrow-band imaging vs. white light. The results showed that compared with white light cystoscope, 5-ALA fluorescence cystoscope could improve RFS (*HR* = 0.65, 95% CI 0.49–0.85), but could not improve PFS (*HR* = 0.63, 95% CI 0.38–1.05); Compared with white light cystoscopy, HAL fluorescent cystoscopy could improve RFS (*HR* = 0.69, 95% CI 0.58–0.82), but could not improve PFS (*HR* = 0.64, 95% CI 0.41–1.00); Compared with white light cystoscopy, narrow-band imaging could improve RFS (*HR* = 0.73, 95% CI 0.60–0.90), but could not improve PFS (*HR* = 0.47, 95% CI 0.22–1.03). (2) We also referred to the EAU guideline [11], NICE guideline [17], and Guidelines for Diagnosis and Treatment of Urology and Andrology in China [7] (Additional file 2: Question 4).

#### Question 5: What are the indications for a repeat TURBT in patients with NMIBC?

Recommendation: For the conditions of incomplete initial TURBT, no muscle tissue in the first resection specimen, high-risk tumors, T1 tumor, G3/high-grade tumor (except CIS), a repeat TURBT operation is recommended. (Evidence level: 1a; Strength of recommendation: Strong).

Implementation consideration: For TaG1/low-grade tumors, even if there is no muscle tissue in the first resected specimen, a repeat TURBT is not an obligated choice.

Evidence summary: We referred to the recommendations from a relevant systematic review, guidelines and monographs. (1) A systematic review [20] about repeat TURBT on NMIBC published in 2018 recruited 1 RCT and 30 non-RCTs, a total of 8409 patients with high-grade Ta or T1 NMIBC. 1) Residual tumor tissues were found in 17–67% of the patients with Ta stage tumor by a repeat TURBT; Residual tumor tissues were found in 20–71% of the patients with T1 stage tumor by repeat TURBT. 2) For Ta stage tumors, the disease recurrence rate was 16% for the patients who received repeat TURBT, and it was 58% in patients without receiving repeat TURBT; For T1 stage tumors, the recurrence rate was 45% in the patients who received repeat TURBT, and that was 49% in patients without receiving repeat TURBT. 3) The tumor progression rates in patients with Ta stage showed no significant difference between patients who received and not received repeat TURBT; For patients with T1 stage, after 26–66 months of follow-up, 5 of the 6 studies showed that the rate of tumor progression in the group without repeat TURBT was higher than that in the group with repeat TURBT. 4) Two studies have shown that repeat TURBT could slightly reduce the overall mortality on the basis of primary TURBT (22–30% vs. 26–36%). (2) We also referred to the EAU guideline [11], AUA guideline [15], CUA guideline [16], NICE guideline [17], NCCN guideline [18], Chinese Expert Consensus on Secondary Resection of Non-muscle-invasive Bladder Cancer [9], and Guidelines for Diagnosis and Treatment of Urology and Andrology in China [7] (Additional file 2: Question 5).

#### Question 6: How long is the recommended interval between the initial and a second TURBT for a NMIBC patient?

Recommendation: A second TURBT should be performed within 4–6 weeks after initial TURBT. (Evidence level: 4; Strength of recommendation: Strong).

Evidence summary: We referred to the recommendations from the EAU guideline [11], AUA guideline [15], CUA guideline [16], NICE guideline [17], NCCN guideline [18], Chinese Expert Consensus on Secondary Resection of Non-muscle-invasive Bladder Cancer [9], and Guidelines for Diagnosis and Treatment of Urology and Andrology in China [7] (Additional file 2: Question 6).

### Section 2: Clinical concerns related to the intravesical chemotherapy

#### Question 7: What are the contraindications to immediate postoperative intravesical chemotherapy?

Recommendation: Immediate post-operative intravesical chemotherapy is contraindicated in the patients with suspected bladder perforation or severe hematuria. (Evidence level: 4; Strength of recommendation: Strong).

Evidence summary: We referred to the recommendations from the EAU guideline [11], AUA guideline [15], CUA guideline [16], NCCN guideline [18], and Guidelines for Diagnosis and Treatment of Urology and Andrology in China [7] (Additional file 2: Question 7).

#### Question 8: Does immediate postoperative intravesical chemotherapy reduce the risk of recurrence of NMIBC?

Recommendation: Except for those with the contraindications to immediate intravesical chemotherapy after operation, all patients with NMIBC should receive immediate postoperative intravesical chemotherapy within 24 h after TURBT. (Evidence level: 1a; Strength of recommendation: Strong).

Evidence summary: We conducted two meta-analyses and referred to the recommendations from relevant systematic reviews, guidelines and monographs. (1) We performed a meta-analysis to compare the efficacy and safety of single intravesical infusion chemotherapy immediately after TURBT with TURBT alone in the treatment of NMIBC. A total of 8 RCTs (10 studies) [21–28] were included, with a total sample size of 1531 cases and a maximum follow-up time of 108 months. Among these studies, two studies included 417 low-risk patients; one study included 219 patients with low and medium risk; one study included 86 high-risk patients; six studies included 1175 patients with unclear risk. Meta-analysis results showed that: in terms of effectiveness, compared with the TURBT alone group, the combination of TURBT and immediate postoperative intravesical infusion chemotherapy group reduced the 1-year recurrence rate (*RR* = 0.75, 95% CI 0.58–0.95), 2-year recurrence rate (*RR* = 0.76, 95% CI 0.63–0.92), 3-year recurrence rate (*RR* = 0.85, 95% CI 0.77–0.93), 4-year recurrence rate (*RR* = 0.78, 95% CI 0.66–0.93) and 5-year recurrence rate (*RR* = 0.91, 95% CI 0.85–0.98); The recurrence risk in the group receiving TURBT combined with immediate single intravesical infusion chemotherapy was 0.64 times of that in TURBT alone group (*HR* = 0.64, 95% CI 0.53–0.76), but there was no significant difference in the progression risk and overall survival between the two groups (*HR* = 0.74, 95% CI 0.42–1.28; *HR* = 0.68, 95% CI 0.37–1.27). In terms of safety, there was no significant difference in the incidence of hematuria (*RR* = 1.91, 95% CI 0.29–12.63), cystitis (*RR* = 3.88, 95% CI 0.63–24.14), fever (*RR* = 2.87, 95% CI 0.12–66.75) and allergy (*RR* = 2.12, 95% CI 0.39–11.43) between the two groups. There was no heterogeneity among the studies included in the above outcome indicators. (2) We performed another meta-analysis to compare the efficacy and safety between immediate single bladder perfusion chemotherapy combined with early maintenance perfusion chemotherapy after TURBT and early maintenance perfusion chemotherapy after TURBT in the treatment of NMIBC, which covered a total of 16 RCTs (18 studies) [29–44], with a total of 1682 patients and a maximum follow-up time of 60 months. Results showed that in terms of effectiveness, TURBT combined with immediate single intravesical infusion chemotherapy after operation reduced the 1-year recurrence rate (*RR* = 0.51, 95% CI 0.35–0.75), 2-year recurrence rate (*RR* = 0.49, 95% CI 0.39–0.63) and 3-year recurrence rate (*RR* = 0.52, 95% CI 0.30–0.88) of NMIBC patients; there was no significant difference in the recurrence-free survival between the two groups (*HR* = 1.80, 95% CI 0.94–3.45). In terms of the safety, there was no statistical difference between the two groups in the incidence of hematuria (*RR* = 1.34, 95% CI 0.59–3.07), bladder irritation (*RR* = 1.26, 95% CI 0.93–1.69), cystitis (*RR* = 0.80, 95% CI 0.22–2.85), fever (*RR* = 0.93, 95% CI 0.14–6.30) and allergy (*RR* = 3.95, 95% CI 0.17–94.52). There was no heterogeneity among the studies included in the above outcome indicators. (3) We also referred to the EAU guideline [11], AUA guideline [15], CUA guideline [16], NICE guideline [17], NCCN guideline [18], and Guidelines for Diagnosis and Treatment of Urology and Andrology in China [7] (Additional file 2: Question 8).

#### Question 9: Do patients with low-risk tumors only need SIC after TURBT?

Recommendation: Only SIC following TURBT is needed for patients with low-risk tumors. (Evidence level: 4; Strength of recommendation: Strong).

Evidence summary: We referred to the recommendations from the EAU guideline [11], AUA guideline [15], CUA guideline [16], NICE guideline [17], NCCN guideline [18], and Guidelines for Diagnosis and Treatment of Urology and Andrology in China[7], summary of recommendations on chemotherapy for NMIBC patients with different risk levels are shown in Additional file 2: Table S1 (Additional file 2: Question 9).

#### Question 10: What are the commonly used drugs and doses for the intravesical chemotherapy?

Recommendation: Drugs, such as gemcitabine, pirarubicin, hydroxycamptothecin, mitomycin-C, doxorubicin and epirubicin, are recommended for intravesical chemotherapy. Under the premise of safe dosage and patients' tolerance, full-dose intravesical chemotherapy is recommended. (Evidence level: 1a; Strength of recommendation: Strong).

Implementation consideration: Commonly used intravesical chemotherapy drugs and their common doses are: Gemcitabine (1000 mg), pirarubicin (30–50 mg), hydroxycamptothecin (10–20 mg), mitomycin-C (20–60 mg), doxorubicin (30–50 mg) and epirubicin (50–80 mg) per usage.

Evidence summary: We conducted 5 meta-analyses and referred to the recommendations from relevant systematic reviews, guidelines and monographs. (1) To compare the efficacy and safety of gemcitabine and pirarubicin for the intravesical infusion chemotherapy of NMIBC, a total of 31 RCTs (32 studies) [45–75] were included in the meta-analysis, with a total of 2182 patients and a maximum follow-up time of 36 months. Results showed that the 1-year recurrence rate (*RR* = 0.58, 95% CI 0.40–0.84), 2-year recurrence rate (*RR* = 0.58, 95% CI 0.48–0.70) and 3-year recurrence rate (*RR* = 0.51, 95% CI 0.31–0.84) of gemcitabine group were lower than those of pirarubicin group, but there was no significant difference in 1-year progression rate (*RR* = 0.60, 95% CI 0.35–1.02) and 3-year progression rate (*RR* = 0.50, 95% CI 0.05–5.19) between the two groups; The recurrence risk in gemcitabine group was 0.47 times of that in pirarubicin group (*HR* = 0.47, 95% CI 0.25–0.89). In terms of safety, the incidence of cystitis (*R*R = 0.35, 95% CI 0.14–0.86), bladder irritation (*RR* = 0.54, 95% CI 0.44–0.65) and hematuria (*RR* = 0.35, 95% CI 0.23–0.52) in gemcitabine group was lower than that in pirarubicin group. There was no significant difference in the incidence of fever (*RR* = 0.77, 95% CI 0.33–1.79) and allergic reaction (*RR* = 0.44, 95% CI 0.17–1.17) between the two groups. There was no heterogeneity among the included studies for the above outcome indicators. Subgroup analysis was carried out according to the dosages and medication schemes. The results showed that the conventional dose of gemcitabine (1000 mg) produced better outcome than the conventional dose group of pirarubicin (30–50 mg) in terms of efficacy indexes (1-year, 2-year and 3-year recurrence rate) and safety indexes (cystitis, bladder irritation sign and hematuria). Under the drug regimen of immediate combined induction and maintenance instillation after TURBT operation in both groups, gemcitabine group saw better outcome than pirarubicin group in terms of efficacy indexes (1-year and 2-year recurrence rate) and safety indexes (cystitis, bladder irritation sign and hematuria) (Additional file 3: Table S1). (2) To compare the efficacy and safety of pirarubicin and hydroxycamptothecin for the intravesical infusion chemotherapy of NMIBC, a total of 14 RCTs [68, 76–88] were included in the meta-analysis, with a total of 1284 patients and a maximum follow-up time of 60 months. Results showed that, in terms of effectiveness, there was no significant difference between the pirarubicin group and the hydroxycamptothecin group in the 1-year recurrence rate (*RR* = 0.79, 95% CI 0.58–1.07), 2-year recurrence rate (*RR* = 0.80, 95% CI 0.61–1.05), 3-year recurrence rate (*RR* = 0.84, 95% CI 0.37–1.91), 4-year recurrence rate (*RR* = 0.92, 95% CI 0.44–1.95) and 5-year recurrence rate (*RR* = 0.73, 95% CI 0.43–1.23). In terms of safety, there was no difference between the two groups in the incidence of total adverse reactions (*RR* = 1.38, 95% CI 0.71–2.70) and hematuria (*RR* = 0.99, 95% CI 0.60–1.62), but the incidence of bladder irritation (*RR* = 1.39, 95% CI 1.14–1.70) in pirarubicin group was higher than that in hydroxycamptothecin group. Except for the indicators of total adverse reaction, there was no heterogeneity among the studies included in the above outcome indicators. Subgroup analysis was carried out according to the dosages and medication schemes. The results showed that the 2-year recurrence rate and the incidence of bladder irritation in the pirarubicin (30–50 mg) group were lower than those in the hydroxycamptothecin (10–20 mg) group. There was no significant difference in these indicators between the two groups under different medication schemes (Additional file 3: Table S1). (3) To compare the efficacy and safety of pirarubicin and mitomycin-C for the intravesical infusion chemotherapy of NMIBC, a total of 25 RCTs [68, 71, 76, 82, 89–109] were included in the meta-analysis, with a total of 2026 patients and a maximum follow-up time of 75 months. Results showed that, in terms of effectiveness, the 1-year recurrence rate (*RR* = 0.50, 95% CI 0.36–0.68) and 2-year recurrence rate (*RR* = 0.45, 95% CI 0.36–0.57) of the pirarubicin group were lower than those of the mitomycin-C group, but there was no significant difference in the 3-year recurrence rate between the two groups (*RR* = 0.66, 95% CI 0.42–1.03). In terms of safety, the incidences of total adverse reactions (*RR* = 0.55, 95% CI 0.42–0.73), bladder irritation sign (*RR* = 0.60, 95% CI 0.48–0.76) and hematuria (*RR* = 0.53, 95% CI 0.30–0.94) in the pirarubicin group were lower than that in the mitomycin-C group. There was no significant difference in the incidence of cystitis between the two groups (*RR* = 0.94, 95% CI 0.37–2.39). Except for the indicators for total adverse reactions and cystitis, there was no heterogeneity among the included studies in other outcome indicators. Subgroup analysis was carried out according to the dosages and medication schemes. The results showed that when comparing the prognosis of patients under different doses of pirarubicin and mitomycin-C, the 2-year recurrence rates in the pirarubicin group were all lower than that in the mitomycin-C group. In the patients treated with induction and maintenance instillation, or combined induction and maintenance instillation immediately after TURBT, the 1-year and 2-year recurrence rates of the pirarubicin group were lower than those of the mitomycin-C group (Additional file 3: Table S1). (4) To compare the efficacy and safety of intravesical instillation chemotherapy with different doses of epirubicin in the treatment of NMIBC, a total of 8 RCTs (12 studies) [110–117] were included in the meta-analysis, with a total of 1114 patients and a maximum follow-up time of 60 months. Results showed that there was no significant difference in the efficacy (1-year and 2-year recurrence rate) and safety (total adverse reaction rate) between the groups of high-dose (> 80 mg) vs. common-dose (50–80 mg), high dose (> 80 mg) vs. low dose (< 50 mg), common dose (50–80 mg) vs. low dose (< 50 mg) of epirubicin in the treatment of NMIBC. (5) To compare the efficacy and safety of intravesical instillation chemotherapy with different doses of pirarubicin in the treatment of NMIBC, 2 RCTs (4 studies) [118, 119] were included in the meta-analysis, with a total of 258 patients and a maximum follow-up time of 38 months. Results showed that the 2-year recurrence rate under pirarubicin treatment (*RR* = 0.35, 95% CI 0.18–0.68; *RR* = 0.44, 95% CI 0.22–0.89) was lower in 50 mg dosage group than that in the 30 mg or 40 mg dosage groups, but the incidence of bladder irritation (*RR* = 1.91, 95% CI 1.16–3.15; *RR* = 2.15, 95% CI 1.19–3.89) was higher in 50 mg pirarubicin group. There was no significant difference in the above indexes between the dose groups of pirarubicin at 40 mg and 30 mg. (6) A network meta-analysis published in 2020 evaluated the efficacy of mitomycin-C, doxorubicin, epirubicin, gemcitabine and thiotepa in the treatment of NMIBC, including 55 RCTs and 12,462 patients [120]. Results showed that compared with TURBT only, except doxorubicin (*HR* = 0.94, 95% CI 0.66–1.35) and cetidipine (*HR* = 0.36, 95% CI 0.10–1.26), the other three chemotherapeutic drugs could reduce the risk of disease progression, with a ranking of gemcitabine > mitomycin-C > epirubicin in their superiority of therapy. Except for cetidipine (*HR* = 0.69, 95% CI 0.41–1.14), the other four chemotherapeutic drugs could reduce the risk of recurrence, with a ranking of gemcitabine > mitomycin-C > epirubicin > doxorubicin in their superiority of therapy. Considering the combined results of recurrence and progression, gemcitabine was the most effective treatment regimen. In the subgroup analysis according to drug regimen, tumor characteristics and literature quality, the results are still stable. (7) We also referred to the EAU guideline [11], NICE guideline [17], NCCN guideline [18], and Guidelines for Diagnosis and Treatment of Urology and Andrology in China [7] (Additional file 2: Question 10).

#### Question 11: How to improve the efficacy of intravesical chemotherapy?

Recommendation: The efficacy of intravesical chemotherapy can be improved by reduction of fluid intake and urine excretion to maintain the drug concentration, instillation for 0.5–2 h (according to the drug instructions), or hyperthermic instillation. (Evidence level: 4; Strength of recommendation: Strong).

Evidence summary: We referred to the recommendations from the EAU guideline [11]and Guidelines for Diagnosis and Treatment of Urology and Andrology in China [7] (Additional file 2: Question 11).

#### Question 12: How to manage the adverse reactions of intravesical chemotherapy?

Recommendation: The adverse reactions of intravesical chemotherapy are related to the dose and frequency of instillation. The most important adverse reactions are chemical cystitis, hematuria, and bladder irritation. If severe bladder irritation occurs, the instillation should be delayed or stopped. Most adverse reactions disappear spontaneously after discontinuation of instillation. (Evidence level: 4; Strength of recommendation: Strong).

Evidence summary: We referred to the recommendations from the Guidelines for Diagnosis and Treatment of Urology and Andrology in China [7]: Chemical cystitis, manifesting as bladder irritation symptoms and hematuria, is the main adverse reaction from intravesical chemotherapy, the severity of which is dependent on the dosage and frequency of instillation. If severe bladder irritation symptoms occur, the instillation should be delayed or stopped to avoid consequential bladder contracture. Most adverse reactions disappear spontaneously after discontinuation of instillation.

### Section 3: Clinical questions related to the intravesical BCG immunotherapy

#### Question 13: What are the contraindications of intravesical BCG immunotherapy?

Recommendation: Intravesical BCG immunotherapy is contraindicated to patients with visible haematuria, symptomatic urinary tract infection, recent history of traumatic catheterization, active tuberculosis, severe immunosuppression (lymphoma, leukemia, steroid hormone application, AIDS, etc.), allergy to BCG, and operations within two weeks of TURBT. (Evidence level: 4; Strength of recommendation: Strong).

Evidence summary: We referred to the recommendations from the EAU guideline [11], NCCN guideline [18], and Guidelines for Diagnosis and Treatment of Urology and Andrology in China [7] (Additional file 2: Question 13).

#### Question 14: Is intravesical BCG immunotherapy prior to intravesical chemotherapy in patients with NMIBC?

Recommendations: (1) For patients with high-risk tumors, intravesical BCG immunotherapy is recommended. (Evidence level: 1a–1b; Strength of recommendation: Strong). (2) For patients with intermediate-risk tumors, intravesical chemotherapy (Evidence level: 1a–1b; Strength of recommendation: Strong) or intravesical BCG immunotherapy (Evidence level: 1a–1b; Strength of recommendation: Weak) is recommended.

Implementation consideration: Treatment schemes for intravesical BCG immunotherapy: starting intravesical BCG instillation within 2–4 weeks after TURBT; The patients should first be given BCG induction instillation for 6–8 weeks (once a week), followed by BCG maintenance instillation for 1–3 years (once for a week for 3 continuous weeks at 3 and 6 months after TURBT), and then repeat the treatment every 6 months (once a week for 3 continuous weeks).

Evidence summary: We conducted a meta-analysis and referred to the recommendations from relevant systematic reviews, guidelines and monographs. The results from different systematic reviews varied due to the heterogeneous patient characteristics, follow-up times, drugs, medication schemes and other factors. However, most of the studies showed that BCG instillation can reduce the risk of tumor recurrence in the patients with high and medium-risk tumors. (1) We conducted a meta-analysis to compare the efficacy and safety between intravesical BCG immunotherapy and intravesical chemotherapy in the treatment of NMIBC. A total of 25 studies (35 studies) [121–145] were included, with a total of 3820 patients with a maximum follow-up time of 80 months. Results showed that in terms of efficacy, the 2-year recurrence rate (*RR* = 0.60, 95% CI 0.38–0.96) of BCG group was lower than that of chemotherapy group, but there was no significant difference in the 3-year recurrence rate (*RR* = 0.69, 95% CI 0.44–1.07), 4-year recurrence rate (*RR* = 1.84, 95% CI 0.72–4.68) and 5-year recurrence rate (*RR* = 0.74, 95% CI 0.50–1.10) between the two groups. There was no significant difference in 1-year disease progression rate (*RR* = 0.69, 95% CI 0.20–2.37), 2-year disease progression rate (*RR* = 0.58, 95% CI 0.20–1.70) and 3-year disease progression rate (*RR* = 0.28, 95% CI 0.06–1.38) between the two groups, but the 5-year disease progression rate in BCG group was lower than that in chemotherapy group (*RR* = 0.58, 95% CI 0.36–0.91). There was no significant difference in the 5-year mortality between the two groups (*RR* = 0.80, 95% CI 0.59–1.08). The recurrence free survival in BCG group was 0.45 times that in chemotherapy group (*HR* = 0.45, 95% CI 0.32–0.63), but there was no significant difference in the overall survival between the two groups (*HR* = 0.90, 95% CI 0.71–1.15). In terms of safety, the incidence of bladder irritation (*RR* = 2.41, 95% CI 1.57–3.69), hematuria (*RR* = 1.80, 95% CI 1.46–2.21), cystitis (*RR* = 2.29, 95% CI 1.72–3.05) and fever (*RR* = 4.70, 95% CI 3.09–7.14) in BCG group were significantly higher than those in chemotherapy group. Except for the 1-year recurrence rate, 4-year recurrence rate and hematuria index, there was no heterogeneity among the studies included in the outcome indicators mentioned above. 1) Subgroup analysis was conducted according to the BCG instillation time (≤ 1 year, > 1 year). When the instillation time was ≤ 1 year, the 2-year and 3-year recurrence rate of BCG group was lower than that of chemotherapy group, but the incidence of bladder irritation, hematuria, cystitis and fever was significantly higher than that of chemotherapy group. When the instillation time was > 1 year, the 5-year recurrence rate of BCG group was lower than that of chemotherapy group, and the safety index results of the two groups remained unchanged (Additional file 3: Table S2). 2) Subgroup analysis was conducted according to the dosage of BCG. When the dosage of BCG was less than 80 mg, the 5-year recurrence rate of BCG group was lower than that of chemotherapy group, but there was no significant difference in the incidence of cystitis and allergy between the two groups. When the dose of BCG was 80–120 mg, the 2-year and 5-year recurrence rate of BCG group was lower than that of chemotherapy group, but the incidence of bladder irritation, hematuria, cystitis and fever were significantly higher than that of chemotherapy group. When the dose of BCG was > 120 mg, the 3-year recurrence rate of BCG group was lower than that of chemotherapy group, and the incidence of hematuria, cystitis and fever was significantly higher than that of chemotherapy group (Additional file 3: Table S2). 3) Subgroup analysis was also conducted according to the BCG instillation scheme. For the induction plus maintenance instillation scheme, the 2-year and 5-year recurrence rate and the 5-year progression rate of BCG group were lower than those of chemotherapy group, but the incidence of bladder irritation, hematuria, cystitis and fever were higher than those of chemotherapy group (Additional file 3: Table S2). 4) Since most of the included studies could not distinguish the risk levels of the included patients, subgroup analysis was not conducted according to the risk level of patients. (2) A network meta-analysis published in 2020 evaluated the efficacies of BCG, mitomycin-C, doxorubicin, epirubicin, gemcitabine and thiotepa in the treatment of NMIBC, including 55 RCTs and 12,462 patients [120]. Results showed that in terms of reducing the risk of recurrence, BCG was better than mitomycin-C, doxorubicin, epirubicin and cetiritin, but there was no statistical difference when compared with gemcitabine. The ranking in reducing the recurrence risk was as follows: Gemcitabine > BCG > mitomycin-C > epirubicin > cetiritin > doxorubicin. In terms of reducing the risk of progression, BCG was superior to doxorubicin and epirubicin, but there was no significant difference when comparing it with gemcitabine, mitomycin-C and cetidipine. The ranking was as follows: gemcitabine > BCG > mitomycin-C > cetidipine > epirubicin > doxorubicin. In the subgroup analysis according to drug regimen, tumor characteristics and literature quality, the results were still stable. (3) A systematic review published by the Cochrane library in 2020 evaluated the efficacy and safety of BCG and mitomycin-C in patients with high-risk Ta and T1 bladder cancer [146]. It included a total of 12 RCTs and 2932 patients. Results showed that there was no significant difference between BCG and mitomycin-C in reduction of all-cause death (*HR* = 0.97, 95% CI 0.79–1.20), tumor recurrence (*HR* = 0.88, 95% CI 0.71–1.09) and tumor progression (*HR* = 0.96, 95% CI 0.73–1.26), and there was no significant difference between them in serious adverse reactions (*RR* = 2.31, 95% CI 0.82–6.52) too. The grade of evidence quality of the above indicators is classified as low level. (4) We also referred to the EAU guideline [11], AUA guideline [15], CUA guideline [16], NICE guideline [17], and Guidelines for Diagnosis and Treatment of Urology and Andrology in China [7] (Additional file 2: Question 14).

#### Question 15: Is a standard dose of BCG immunotherapy superior to a low dose of BCG immunotherapy for the patients with intermediate-risk and high-risk NMIBC?

Recommendations: (1) For patients with high-risk tumors, a standard dose of BCG immunotherapy is recommended. (Evidence level: 1a; Strength of recommendation: Strong). (2) For patients with intermediate-risk tumors, the recommended dose of BCG immunotherapy shall not be less than 1/3 of the standard dose. (Evidence level: 1b; Strength of recommendation: Weak).

Implementation consideration: The standard dose of BCG can be 80–120 mg according to the patient's clinical condition, such as the stage, grade, diameter and number of tumors.

Evidence summary: We conducted one meta-analysis and referred to the recommendations from relevant systematic reviews, guidelines and monographs. (1) We conducted a meta-analysis to compare the efficacy and safety of low-dose BCG and standard dose BCG in the treatment of NMIBC [147]. A total of 13 RCTs (18 studies) were included, with a total of 2903 patients and the maximum follow-up time of 84 months. Results showed that in terms of efficacy, the recurrence risk was higher in low dose BCG treated patients than that in the standard dose GCG group (*HR* = 1.21, 95% CI 1.06–1.39), but there was no significant difference in the risk of progression between the groups with the two dosages (*HR* = 1.09, 95% CI 0.86–1.38). In terms of safety, the incidence of total adverse reactions (*RR* = 0.64, 95% CI 0.51–0.80), systemic adverse reactions (*RR* = 0.67, 95% CI 0.47–0.95) and serious adverse reactions (*RR* = 0.51, 95% CI 0.35–0.73) in the low-dose BCG group were lower than those in the standard-dose BCG group, but there was no significant difference in the incidence of local adverse reactions (*RR* = 0.92, 95% CI 0.79–1.07) between the two groups. There was no heterogeneity for the above efficacy indicators among the studies, but heterogeneity existed in the safety indicators among the studies included. 1) Subgroup analysis was performed according to the instillation scheme (simple induction, induction plus maintenance) (Additional file 3: Table S3). The analysis results showed that the efficacy outcomes of the two groups were consistent with the above conclusions, and the overall adverse reactions of the low-dose BCG group were less than that of the standard dose BCG group. Because the included literature mostly did not demonstrate the risk levels of the patients, subgroup analysis was not performed according to the risk level of the patients. 2) Among the included studies, the results of an RCT [148] (*n* = 1349) showed that, for patients with high-risk tumors (multiple tumors, recurrence times ≥ 2, G3, CIS), the standard dose of BCG was advantageous over the 1/3 standard dose treatment in reducing the risk of disease recurrence. For patients with intermediate-risk tumors, there was no significant difference in reducing the risk of disease recurrence between the standard-dose and 1/3 standard-dose BCG groups. 3) The low doses of BCG used in these included studies were 27 mg, 40 mg, 45 mg and 60 mg, and the standard doses were 80 mg, 81 mg, 90 mg, 100 mg and 120 mg. In the meta-analysis conducted by the evidence synthesis group, the low dose of BCG was defined as < 80 mg, and the standard dose of BCG was defined as 80–120 mg. 4) According to the conclusions of the meta-analysis, it is suggested to stratify the recommendations per the risk levels of the patients, that is, standard dose of BCG immunotherapy is recommended for high-risk patients and low-dose BCG perfusion therapy is recommended for intermediate-risk patients. (2) We also referred to the EAU guideline [11], NCCN guideline [18], and Guidelines for Diagnosis and Treatment of Urology and Andrology in China [7] (Additional file 2: Question 15).

#### Question 16: Is BCG induction plus its maintenance instillation superior to BCG induction instillation alone in the patients with NMIBC?

Recommendation: BCG induction plus its maintenance instillation is recommended for NMIBC patients with intermediate-risk and high-risk tumors. (Evidence level: 1a; Strength of recommendation: Strong).

Implementation consideration: The instillation scheme generally includes 6–8 weeks (once a week) of BCG induction instillation within 2–4 weeks after TURBT, and 1–3 years of BCG maintenance instillation. The 1-year maintenance instillation scheme can be considered: after the induction instillation, start the intensive instillation once 2 weeks for a total of 3 continuous times, followed by maintenance instillation once a month for a total of 10 times. For the patients with high-risk tumors, a 3-year maintenance instillation scheme can be considered: after induced instillation, start the continuous instillation for 3 weeks periodically at the 3rd, 6th, 12th, 18th, 24th, 30th and 36th months respectively.

Evidence summary: We conducted a meta-analysis and referred to the recommendations from relevant systematic reviews, guidelines and monographs. (1) We conducted the meta-analysis to compare the efficacy and safety of BCG induction plus maintenance instillation and induction instillation alone in NMIBC patients [149]. Thirteen RCTs were included, with a total of 1625 patients and a maximum follow-up time of 120 months. Results showed that, in terms of efficacy, compared with BCG induction instillation alone, BCG induction plus maintenance instillation reduced the recurrence risk (*HR* = 0.53, 95% CI 0.42–0.65), progression risk (*HR* = 0.72, 95% CI 0.53–0.90) and overall rate of tumor recurrence and progression (*RR* = 0.78, 95% CI 0.62–0.98) In terms of safety, the incidences of frequent urination, dysuria, urinary pain, gross hematuria, fever and fatigue in the BCG induction plus maintenance group were higher than that in the simple induction group. There was no heterogeneity among the included studies for the indicators of recurrence risk and progression risk, but there was heterogeneity among the studies in the indicators of overall rate of recurrence and progression. 1) Among the 13 RCTs included, 10 RCTs were studied in NMIBC patients with intermediate-risk or high-risk tumors. When analysis was conducted for these patients with intermediate-risk or high-risk tumors, the results showed that compared with BCG induction instillation alone, BCG induction plus maintenance instillation reduced the recurrence risk, progression risk and the overall rate of recurrence and progression (Additional file 3: Table S4). 2) Among the 13 RCTs included, 6 RCTs focused on NMIBC patients with high-risk tumors. When analysis was conducted for these patients with high-risk tumors, the results showed that compared with BCG induction instillation alone, BCG induction plus maintenance instillation reduced the recurrence risk and the overall rate of recurrence and progression, but there is no significant difference in the progression risk (Additional file 3: Table S4). 3) Subgroup analysis was performed according to the time of maintenance instillation (< 2 years, ≥ 2 years) and BCG dosages (≤ 81 mg, > 81 mg) (Additional file 3: Table S4). The results showed that no matter whether the maintenance perfusion time was ≥ 2 years or < 2 years, the induction plus maintenance instillation had an advantage over simple induction instillation in reducing the recurrence risk. In the patients with the maintenance instillation dose ≤ 81 mg, the induction plus maintenance instillation had advantages over simple induction instillation in reducing the recurrence risk, the progression risk, and the overall rate of recurrence and progression. (2) We also referred to the EAU guideline [11], AUA guideline [15], CUA guideline [16], NICE guideline [17], and Guidelines for Diagnosis and Treatment of Urology and Andrology in China [7] (Additional file 2: Question 16).

#### Question 17: For the patients with NMIBC, is the 3-year BCG maintenance instillation superior to the 1-year maintenance instillation?

Recommendations: (1) For patients with high-risk tumors, standard dose of BCG maintenance instillation for 1–3 years is recommended. (Evidence level: 1b; Strength of recommendation: Strong). (2) The patients with intermediate-risk tumors can be treated with BCG maintenance instillation for 1 year. The final choice should be made by comprehensively taking into account the risk of recurrence and progression, adverse reactions and the medical conditions of patients. (Evidence level: 1b; Strength of recommendation: Strong).

Implementation consideration: The 3-year maintenance instillation scheme can be as follows: induction instillation for 6–8 weeks after TURBT (once/week), and then followed by the maintenance instillation for 3 weeks at the 3rd, 6th, 12th, 18th, 24th, 30th and 36th months. The 1-year maintenance instillation scheme can be the following: induction instillation for 6–8 weeks (once/week), followed by intensive instillation once 2 weeks for a total of 3 continuous times, and then maintenance instillation once a month for a total of 10 times.

Evidence summary: We referred to the recommendations from relevant systematic reviews, guidelines and monographs. (1) An RCT published in 2013 included 1355 patients and compared the therapeutic effects of 1-year maintenance instillation and 3-year maintenance instillation [148]. The results showed that the 5-year recurrence-free rates resulted from the 1-year and 3-year maintenance instillation were 56.6% and 63.4% respectively. In the high-risk patients who received standard-dose treatment, the 3-year maintenance instillation further reduced the recurrence risk compared with the 1-year maintenance instillation (*HR* = 0.62, 95% CI 0.43–0.88). For the intermediate-risk patients who received 1/3 standard-dose treatment, the 3-year maintenance instillation reduced the recurrence risk compared with the 1-year maintenance instillation (*HR* = 0.74, 95% CI 0.56–0.97). (2) We also referred to the EAU guideline [11], AUA guideline [15], CUA guideline [16], and NCCN guideline [18] (Additional file 2: Question 17).

#### Question 18: What is the treatment option after the intravesical BCG immunotherapy failed?

Recommendations: (1) For the patients with high-risk recurrences, (T1 stage, CIS, or high-grade tumor), radical cystectomy is recommended. (Evidence level: 4; Strength of recommendation: Strong). (2) For the patients with high-risk recurrences but not suitable for or refuse a radical cystectomy, bladder preservation strategies (comprehensive treatment, intravenous chemotherapy, etc.) can be offered. (Evidence level: 1b; Strength of recommendation: Weak).

Implementation consideration: Recurrence of low-risk tumors after BCG instillation is not considered treatment failure, and BCG instillation can be offered again. The treatment option for the recurrence of intermediate-risk tumors is between the intensity of options for low-risk and high-risk recurrence. The actual choice is to be determined through full discussion between doctors and patients.

Evidence summary: We referred to the recommendations from relevant systematic reviews, guidelines and monographs. (1) A systematic review published in 2020 evaluated the efficacy of bladder-sparing therapies after BCG treatment failure, including 4 randomized controlled trials and 24 single arm studies [150]. The results showed that the 12-month response rates were 24% for the patients who received two or more prior courses of BCG and 36% for those who received one or more prior courses of BCG. (2) A systematic review published in 2020 including 42 studies with 24 treatment regimens and 2254 patients with NMIBC [151] also evaluated the efficacy of bladder-preserving therapies after BCG treatment failure. The results showed that the bladder-sparing treatments produced a median complete response rate of 26% at 6 months, 17% at 12 months and 8% at 24 months in the patients with CIS. In contrast, they produced the median recurrence-free rate of 67% at 6 months, 44% at 12 months and 10% at 24 months in the patients with bladder papilloma. (3) We also referred to the EAU guideline [11], AUA guideline [15], CUA guideline [16], NICE guideline [17], and Guidelines for Diagnosis and Treatment of Urology and Andrology in China[7] (Additional file 2: Question 18; Additional file 2: Tables S2, S3).

#### Question 19: How to manage the side effects of intravesical BCG immunotherapy?

Recommendation: The side effects of intravesical BCG immunotherapy include local and systemic side effects, and the corresponding managements are shown in Table 2. (Evidence level: 4; Strength of recommendation: Strong).

**Table 2:** Management of BCG side effects

Local side effect	Management
Symptoms of cystitis	If symptoms are mild, drugs for relieving bladder irritation (e.g., finapyridine), anticholinergic, and non-steroidal anti-inflammatory are feasible. Continue the instillations when symptoms improve within a few days
	If symptoms persist (> 48 h) or worsen: (1) Postpone the instillation or reduce the dose of BCG (2) Perform a urine culture (3) Start empirical antibiotic treatment (e.g., oral quinolone antibiotics)
	If symptoms persist after antibiotic treatment: (1) Postpone the instillation (2) With positive culture: adjust antibiotic treatment according to sensitivity (3) With negative culture: intravesical instillation therapy with quinolones and anti-inflammatory and analgesic drugs, once daily for 5 d (repeat if necessary)
	If symptoms persist, treat with oral anti-tuberculosis drugs (e.g., isoniazid, rifampicin) and corticosteroids
	If there is no response to the treatment and/or caused severe contracted bladder, perform radical cystectomy when necessary
Haematuria	Perform urine culture to exclude haemorrhagic cystitis, if other symptoms present. Perform the instillation again when the urine is clear
	If haematuria persists, perform cystoscopy to evaluate the presence of bladder tumor
	If macro-hematuria occurs, indwelling catheter and continuous bladder irrigation are recommended, and perform endoscopic hemostasis treatment if necessary
Granulomatous prostatitis	If symptoms present, perform urine culture, suspend the instillation, and give isoniazid and rifampicin orally for three months, plus quinolone antibiotics and cortisol drugs. Asymptomatic patients do not require any treatment
Epididymo-orchitis	Perform urine culture, cease intravesical therapy, administer quinolone antibiotics or anti-tuberculous drugs. If symptoms persist, hormone therapy is feasible. Abscess incision drainage is also feasible when abscess occurs. If the treatments above are not effective, consider orchiectomy when necessary
Urethral stricture	Postpone the instillation, perform spasmolytic treatment. Continue the instillations when symptoms are relieved within a few days, and avoid drugs flowing into urethra during instillations. If the symptoms persist or worsen, urethral dilatation or urethrotomy is feasible
Bladder contracture	Postpone the instillation, use lidocaine for sedation and analgesia, perform bladder enlargement if necessary

Systemic side effect	Management
General malaise/fever	Observation for the patients with mild symptoms which resolve within 48 h
	If symptoms worsen (> 38.5 °C for > 48 h), suspend BCG instillations, perform urine culture for bacteria and acid-fast bacilli, treat the patients with broad-spectrum antibiotics and anti-tuberculosis drugs, and consult with relevant physicians if necessary
BCG sepsis	Strictly follow the contraindications to BCG instillations. BCG should be started at least 2 weeks away after TURBT. When sepsis occurs, stop the BCG treatment immediately, transfer the patients to ICU for treatment, perform urine culture for bacteria and acid-fast bacilli, administer broad-spectrum antibiotics, anti-tuberculosis and hormone drugs. For severe cases without renal failure, consider giving oral cycloserine and strengthening the monitoring of its blood concentration. BCG instillation is no longer recommended after the patient's condition improves
Allergic reactions	(1) Postpone the instillations, or suspend the instillations if symptoms worsen (2) Administer antihistamines and anti-inflammatory agents, and increase the dosage of antibiotics or utilize the anti-tuberculosis drugs, if necessary
Other rare adverse reactions	Most rare adverse reactions are considered to be autoimmune reactions such as arthritis, hepatitis, pneumonia, bone marrow suppression, etc. Non-steroidal anti-inflammatory, cortisol, quinolones or anti-tuberculosis drugs are feasible

*BCG* Bacillus Calmette–Guérin, *TURBT* transurethral resection of bladder cancer, *ICU* Intensive Care Unit

Evidence summary: We referred to the recommendations from the EAU guideline [11], CUA guideline [16], and Guidelines for Diagnosis and Treatment of Urology and Andrology in China [7] (Additional file 2: Question 19; Additional file 2: Tables S4, S5).

### Section 4: Clinical concerns related to the combination therapy

#### Question 20: For the patients with NMIBC, is the combination therapy (intravesical BCG immunotherapy combined with intravesical chemotherapy) superior to the intravesical BCG immunotherapy alone?

Recommendation: For the BCG-unresponsive patients who are not suitable or refuse radical cystectomy, intravesical BCG immunotherapy combined with intravesical chemotherapy can be considered. (Evidence level: 1a–1b; Strength of recommendation: Weak).

Implementation consideration: The following treatment scheme can be considered for intravesical BCG instillation combined with intravesical chemotherapy: Single postoperative instillation of intravesical chemotherapy should be offered immediately after TURBT. Next, the patients should be given maintenance chemotherapy 2–3 times (once a week) followed by BCG instillation immunotherapy (80–120 mg) 2–3 times (once a week), which should be started within 2–3 weeks after TURBT. Then the above intravesical chemotherapy and intravesical BCG instillation should be given alternately for a total of 2–3 months. After that, intravesical chemotherapy and intravesical BCG instillation are still offered alternately but the frequency should be changed to once a month. The whole course of combined treatment lasts for 10–12 months.

Evidence summary: We conducted a meta-analysis and referred to the recommendations from relevant systematic reviews, guidelines and monographs. (1) We conducted a meta-analysis to compare the efficacy and safety of BCG combined chemotherapy (combined group) and BCG alone (BCG group) in the treatment of NMIBC [152]. A total of 13 RCTs were included, with a total of 1754 patients and a maximum follow-up time of 121 months. Results showed that, compared with BCG group, the combined group improved the recurrence-free survival (*HR* = 0.53, 95% CI 0.43–0.66), overall survival (*HR* = 0.66, 95% CI 0.50–0.86) and disease-specific survival (bladder cancer) (*HR* = 0.48, 95% CI 0.29–0.80), but there was no significant difference in the progression-free survival between the two groups (*HR* = 0.65, 95% CI 0.25–1.68). In terms of safety, the incidence of gastrointestinal reactions (*RR* = 2.54, 95% CI 0.61–10.60), cystitis (*RR* = 0.67, 95% CI 0.29–1.54) in the combined group were not significantly different from those in the BCG group. The incidence of fever (*RR* = 0.50, 95% CI 0.27–0.91), bladder irritation (*RR* = 0.69, 95% CI 0.52–0.90) and hematuria (*RR* = 0.50, 95% CI 0.28–0.89) were significantly decreased in the combined group compared to those in the BCG group. There was no heterogeneity among the studies for the above outcome indicators. 1) Subgroup analysis was conducted according to the geographical locations of the studies (China, non-China), chemotherapy drugs used in the combined group (pirarubicin, mitomycin-C, epirubicin), maintenance treatment duration (≤ 1 year, > 1 year), and the facts whether patients were encountered with the complication of CIS (with CIS and without CIS). The conclusion was consistent with the above results, that is, the combined treatment was more advantageous in reducing the risk of recurrence and progression than the BCG group alone. 2) Due to the data limitation, the comparative effects of different courses and frequencies of the combined therapy and the comparative effects of different chemotherapeutic drugs in the combined therapy could not be analyzed. 3) Considering the toxic reactions of BCG, and the facts that most of the patients included in this meta-analysis were intermediate-risk or high-risk patients, the latter making up more than half of the total samples, which limited the feasibility of the conclusion in low-risk patients. Therefore, the combination therapy is not recommended for the non BCG-unresponsive patients. (2) We also referred to the EAU guideline [11] and CUA guideline [16] (Additional file 2: Question 20).

### Section 5: Clinical concerns related to the treatment of NMIBC CIS

#### Question 21: Is intravesical BCG immunotherapy superior to intravesical chemotherapy in the patients with CIS?

Recommendation: For the patients with CIS, the intravesical BCG immunotherapy is recommended after TURBT. (Evidence level: 1a–1b; Strength of recommendation: Strong).

Implementation consideration: For the patients with CIS who underwent radical cystectomy, intravesical BCG immunotherapy is not required. Intravesical BCG instillation usually starts 2–3 weeks after TURBT. Firstly, 6 weeks of induction instillation (once a week) is to be offered. Then those who are responsive to the induction instillation should be given the maintenance instillation for 1–3 years (continuous instillation for 3 weeks at the 3rd, 6th, 12th, 18th, 24th, 30th and 36th months).

Evidence summary: We conducted a meta-analysis and referred to the recommendations from relevant systematic reviews, guidelines and monographs. (1) We conducted a meta-analysis to compare the efficacy and safety of intravesical BCG immunotherapy and intravesical chemotherapy in the treatment of NMIBC CIS patients. A total of 9 RCTs (11 studies) [121, 123, 126, 131, 153–157] were included, with a total of 1231 patients and a maximum follow-up time of 148.8 months. The 11 studies included 1333 patients with high-risk tumors. Results showed that, in terms of effectiveness, intravesical BCG immunotherapy significantly reduced the 72-month recurrence rate (*RR* = 0.70, 95% CI 0.56–0.89) and the 143-month recurrence rate (*RR* = 0.18, 95% CI 0.05–0.72) compared with intravesical chemotherapy, but there was no significant difference between the two groups in the 12-month recurrence rate, 22–69-month and 143–148.8-month progression rate and 143–148.8-month mortality. In terms of safety, the incidence of dysuria (*RR* = 2.25, 95% CI 1.19–4.22), local adverse reactions (*RR* = 3.20, 95% CI 1.23–8.34) and systemic adverse reactions (*RR* = 12.00, 95% CI 1.60–90.23) in BCG instillation group were significantly higher than those in intravesical chemotherapy group, but there was no significant difference in the incidence of fever, hematuria and cystitis. There was no heterogeneity among the studies for the above outcome indicators. As the included studies adopted different follow-up duration and the number of studies included in the meta-analysis of these indicators were limited, the results should be interpreted with caution. (2) We also referred to the EAU guideline [11], AUA guideline [15], CUA guideline [16], NICE guideline [17], NCCN guideline [18], and Guidelines for Diagnosis and Treatment of Urology and Andrology in China [7] (Additional file 2: Question 21).

### Section 6: Clinical concerns related to the radical cystectomy (RC) for NMIBC

#### Question 22: What are the indications for RC in NMIBC patients?

Recommendation: RC is recommended for the patients with high-risk of disease progression. (Evidence level: 4; Strength of recommendation: Strong).

Implementation consideration: Patients with a high risk of disease progression include those who have: 1) HG T1 disease with variant histology (e.g., micropapillary, plasmacytoid, sarcomatoid) or CIS; 2) HG T1 tumor with lymphovascular invasion, multiple and/or large HG T1 tumors, and HG T1 tumors with CIS of bladder/prostate; 3) BCG-unresponsive tumors; 4) presence of persistent or recurrent HG T1 tumor after TURBT; 5) presence of large (≥ 3 cm), diffuse, endoscopically unresectable tumors.

Evidence summary: We referred to the recommendations from the EAU guideline [11], AUA guideline [15], CUA guideline [16], NCCN guideline [18], and Guidelines for Diagnosis and Treatment of Urology and Andrology in China [7] (Additional file 2: Question 22).

### Section 7: Clinical concerns related to the treatment of NMIBC recurrence

#### Question 23: Is intravesical BCG immunotherapy superior to intravesical chemotherapy in patients with recurrent NMIBC?

Recommendations: (1) For the patients with recurrent low-risk tumors and small papilloma after intravesical chemotherapy, a single intravesical chemotherapy instillation can be given. (Evidence level: 4; Strength of recommendation: Weak). (2) For the patients with recurrent low-risk tumors after intravesical BCG immunotherapy, continued intravesical BCG immunotherapy can be considered. (Evidence level: 4; Strength of recommendation: Weak). (3) For the patients with recurrent high-risk tumors, except for those who need RC, intravesical BCG instillations are recommended. (Evidence level: 4; Strength of recommendation: Strong).

Implementation consideration: For the patients with recurrent low-risk tumors and small papilloma after intravesical chemotherapy, a single immediate instillation of intravesical chemotherapy can be administered. Currently, there is no evidence supporting the need of intravesical chemotherapy maintenance, and it is still controversial whether to perform single immediate instillation of chemotherapy alone. The treatment schedule for medium-risk recurrent tumors is between the aggressivity of low-risk and high-risk recurrent diseases, and the final choice can be determined by the discussion between doctors and patients.

Evidence summary: We referred to the recommendations from the EAU guideline [11], AUA guideline [15], CUA guideline [16], and Guidelines for Diagnosis and Treatment of Urology and Andrology in China [7] (Additional file 2: Question 23).

### Section 8: Clinical concerns related to the follow-up of patients with NMIBC

#### Question 24: How to manage the follow-up for the NMIBC patients after TURBT?

Recommendations: (1) For the patients with low-risk tumors, the first cystoscopy should be performed in the third month after TURBT, followed by a cystoscopy in the 12th month, and then cystoscopy should be performed once a year from the second to the fifth years. (Evidence level: 4; Strength of recommendation: Strong). (2) For the patients with intermediate-risk tumors, the first cystoscopy and urine cytology should be performed in the third month after TURBT, followed by tests of cystoscopy and urine cytology in the 6th, 9th, 12th, 18th and 24th months. Then cystoscopy and urine cytology should be performed once a year from the third to fifth years. In the first year, baseline examinations by upper urography, abdominal and pelvic imaging should be conducted. (Evidence level: 4; Strength of recommendation: Strong). (3) For the patients with high-risk tumors, cystoscopy and urine cytology should be performed every 3 months after TURBT. From the third to fifth years, cystoscopy and urine cytology should be performed once every six months. After the 6th year, cystoscopy and urine cytology should be performed once a year. Baseline examinations of upper urinary tract imaging and imaging of chest, abdomen and pelvis should be conducted within the first year. Additionally, the upper urinary tract imaging examination should also be performed at the 12th month and every 1–2 years after that for 10 years. (Evidence level: 4; Strength of recommendation: Strong). (4) Integrated dual-channel bladder catheter is recommended for the cystoscopy, which is effective to prevent potential infection and reduce the damage of cystoscope. (Evidence level: 1b; Strength of recommendation: Weak).

Implementation consideration: Upper urography examinations include: CT urography, MR urography, intravenous urography, ascending urography or ureteroscopy. Baseline examination represents that performed in the perioperative period. If it is performed before TURBT, it does not need to be repeated within 1 year after TURBT.

Evidence summary: We referred to the relevant clinical trials and recommendations from relevant systematic reviews, guidelines. (1) The evidence synthesis group conducted a randomized, open-labeled controlled trial to compare the efficacy and safety of integrated dual-channel cystoscopy in 140 Chinese patients (Clinical Trial Registration No.: chictr180014256). The results showed that the integrated dual-channel bladder catheter was equipped with high-definition camera, and well-sealed and connected to the cystoscope. It was convenient and comfortable for doctors to operate [6]. (2) We also referred to the EAU guideline [11], AUA guideline [15], CUA guideline [16], NICE guideline [17], and NCCN guideline [18]. The relevant recommendations in the existing guidelines are shown in Additional file 2: Table S6 (Additional file 2: Question 24).

## Discussion

During this guideline development, we adopted rigorous search techniques for the evidence synthesis and guideline development methodology was strictly followed for the formulation of recommendations. The medical resources and conditions in the primary medical institutions were also taken into account to justify the recommendations. Meanwhile, attention should be paid to the following points in the future research or update of future guidelines: (1) Many clinical managements lack the support from research evidence or high-quality research evidence, such as the treatment measures of recurrent NMIBC. Meanwhile, the difference in the tumor characteristics, adopted drugs and drug regimens raised heterogeneity among the research results. Thus, high-quality clinical trials are needed to provide solid comparable evidence. (2) To meet the medical resources and conditions in primary medical institutions, researches conducted in the primary medical institutions can provide more appropriate evidence than studies conducted in other medical institutions for the recommendations targeting NMIBC patients in these institutions. However, few studies we searched met this condition. (3) The evidence from health economics is the factor to be considered in determining the recommendations, as economic burden is an important factor that influences the decision-making of the patients in primary medical institutions. However, at present, the economic evidence related to the use of TURBT, chemotherapy and immunotherapy in NMIBC patients is still lacking, such as the research on comparative health economics between TURBT and other surgical treatment devices, and that between chemotherapy and BCG immunotherapy, etc. (4) The anatomical proportion of Chinese people is different from that of European and American people. The comfort and adaptability of the imported TURBT equipment for the patients need to be studied. Additionally, it is valuable to compare the effect and safety of TURBT equipment in the treatment of NMIBC between domestic equipment and imported equipment to refine the application settings of medical devices. (5) Currently, there is little evidence on the application of immune checkpoint-targeting therapies such as PD-1/PD-L1-targeting therapy in the NMIBC patients, which is a new branch of applications to be investigated other than the intravesical chemotherapy and BCG immunotherapy. (6) Patient values/preferences are one of the three elements of evidence-based medicine. The development of this guideline did not investigate the influence of patient values/preferences, which also needs to be taken into account in the future.

## Supplementary Information

**Additional file 1**. Classification criterion of NMIBC

**Additional file 2**. Summary of recommendations from relevant guidelines and monographs

**Additional file 3**. Results of meta-analyses

## Data Availability

All data generated or analyzed in this study are included in this published article and its supplementary information files.

## Abbreviations

5-ALA: 5-aminolevulinic acid
AUA: American Urological Association
BCG: Bacillus Calmette–Guérin
CIS: Carcinoma in situ, tumor in situ
CUA: Canadian Association of Urology
CUETO: Club Urologico Español de Tratamiento Oncologico
EAU: European Association of Urology
EMDA: Electromotive drug administration
EORTC: European Organisation for Research and Treatment of Cancer
HAL: Hexaminolevulinate
HG: High grade
INF: Interferon
LG: Low grade
LVI: Lymphovascular invasion
MIBC: Muscle-invasive bladder cancer
NMIBC: Non-muscle-invasive bladder cancer
NCCN: National Comprehensive Cancer Network
NICE: National Institute for Health and Care Excellence
OS: Overall survival
PFS: Progression-free survival
RC: Radical cystectomy
RFS: Recurrence-free survival
RCT: Randomized controlled trial
TURBT: Transurethral resection of bladder cancer
SIC: Single postoperative instillation of intravesical chemotherapy
SWOG: South West Oncology Group

## References

[1] Lenis AT, Lec PM, Chamie K, Mshs MD (2020). Bladder cancer: a review. JAMA.

[2] Richters A, Aben KKH, Kiemeney L (2020). The global burden of urinary bladder cancer: an update. World J Urol.

[3] Zi H, He SH, Leng XY, Xu XF, Huang Q, Weng H (2021). Global, regional, and national burden of kidney, bladder, and prostate cancers and their attributable risk factors, 1990–2019. Mil Med Res.

[4] Kamat AM, Hahn NM, Efstathiou JA, Lerner SP, Malmström PU, Choi W (2016). Bladder cancer. Lancet.

[5] Taylor J, Becher E, Steinberg GD (2020). Update on the guideline of guidelines: non-muscle-invasive bladder cancer. BJU Int.

[6] Urological Association of Chinese Research Hospital Association (Crha), Urological Health Promotive Association of China International Exchange and Promotive Association for Medical and Health Care (Cpam), Evidence-Based Medicine Association of China International Exchange and Promotive Association for Medical and Health Care (Cpam), The Project Team for Minimally Invasive Plasma Kinetic System and Cloud Planning Solution of the National Key Research and Development Program of China. Treatment and surveillance for non-muscle-invasive bladder cancer in China: an evidence-based clinical practice guideline (2018 standard version). J Modern Urol. 2019;24(7):516–42.

[7] Guo YL, Na YQ, Ye ZQ, Huang J (2019). Guidelines for diagnosis and treatment of urology and andrology in China.

[8] Li CL (2011). Standardization of cancer diagnosis and treatment series—bladder cancer and prostate cancer.

[9] Chinese Urological Association of Chinese Medical Association, Chinese Bladder Cancer Consortium. Chinese consensus statements of second transurethral resection for non-muscle invasive bladder cancer. Chin J Urol. 2017;38(8):561–3.

[10] Ma LL, Wang YY, Yang ZH, Huang D, Weng H, Zeng XT (2020). Methodological quality (risk of bias) assessment tools for primary and secondary medical studies: what are they and which is better?. Mil Med Res.

[11] EAU. EAU-ESTRO-SIOG Guidelines on Non-muscle-invasive Bladder Cancer. https://uroweb.org/guideline/non-muscle-invasive-bladder-cancer/.

[12] Brierley J, Gospodarowicz M, Wittekind C. TNM classification of malignant tumors. UICC International Union Against Cancer. 8th ed. New York: Wiley-Blackwell and UICC; 2016.

[13] Moch H, Humphrey Pa, Ulbright Tm, Ve R. WHO Classification of Tumours of the Urinary System and Male Genital Organs, 4th ed. 2016. https://publications.iarc.fr/Book-And-Report-Series/Who-Classification-Of-Tumours/WHO-Classification-Of-Tumours-Of-The-Urinary-System-And-Male-Genital-Organs-2016. 10.1016/j.eururo.2016.02.028 26996659

[14] Mostofi F, Sorbin L, Torloni H. Histologic typing of urinary bladder tumours. International classification of tumours, No 10. Geneva: WHO; 1973.

[15] Chang S, Bochner B, Chou R, Clark P. Diagnosis and Treatment of Non-Muscle Invasive Bladder Cancer: AUA/SUO Joint Guideline (2020). https://www.auanet.org/guidelines/guidelines/bladder-cancer-non-muscle-invasive-guideline.

[16] Bimal B, Ronald K, Girish S, Siemens D, Armen G. Canadian Urological Association guideline on the management of non-muscle invasive bladder cancer 2021. https://www.cua.org/system/files/Guidelines/7367_NMIBC%20Guideline_Epub.pdf.

[17] National Institute for Health and Care Excellence (Nice). Bladder cancer: diagnosis and management. https://www.nice.org.uk/guidance/ng2. 26180884

[18] National Comprehensive Cancer Network (Nccn). NCCN clinical practice guidelines in oncology: Bladder cancer. 2022. https://www.nccn.org/guidelines/guidelines-detail?category=1&id=1417.

[19] Li H, Cao Y, Ma P, Ma Z, Li C, Yang W (2021). Novel visualization methods assisted transurethral resection for bladder cancer: an updated survival-based systematic review and meta-analysis. Front Oncol.

[20] Cumberbatch MGK, Foerster B, Catto JWF, Kamat AM, Kassouf W, Jubber I (2018). Repeat transurethral resection in non-muscle-invasive bladder cancer: a systematic review. Eur Urol.

[21] Ali-el-Dein B, Nabeeh A, el-Baz M, Shamaa S, Ashamallah A. Single-dose versus multiple instillations of epirubicin as prophylaxis for recurrence after transurethral resection of pTa and pT1 transitional-cell bladder tumours: a prospective, randomized controlled study. Br J Urol. 1997;79(5):731–5. 10.1046/j.1464-410x.1997.00142.x 9158511

[22] Barghi MR, Rahmani MR, Hosseini Moghaddam SM, Jahanbin M (2006). Immediate intravesical instillation of mitomycin C after transurethral resection of bladder tumor in patients with low-risk superficial transitional cell carcinoma of bladder. Urol J.

[23] The effect of intravesical thiotepa on tumour recurrence after endoscopic treatment of newly diagnosed superficial bladder cancer. A further report with long-term follow-up of a Medical Research Council randomized trial. Medical Research Council Working Party on Urological Cancer, Subgroup on Superficial Bladder Cancer. Br J Urol. 1994;73(6):632–8. 10.1111/j.1464-410x.1994.tb07547.x 8032829

[24] De Nunzio C, Carbone A, Albisinni S, Alpi G, Cantiani A, Liberti M (2011). Long-term experience with early single mitomycin C instillations in patients with low-risk non-muscle-invasive bladder cancer: prospective, single-centre randomised trial. World J Urol.

[25] Gudjónsson S, Adell L, Merdasa F, Olsson R, Larsson B, Davidsson T (2009). Should all patients with non-muscle-invasive bladder cancer receive early intravesical chemotherapy after transurethral resection? The results of a prospective randomised multicentre study. Eur Urol.

[26] Messing EM, Tangen CM, Lerner SP, Sahasrabudhe DM, Koppie TM, Wood DP (2018). Effect of intravesical instillation of gemcitabine vs saline immediately following resection of suspected low-grade non-muscle-invasive bladder cancer on tumor recurrence: SWOG S0337 randomized clinical trial. JAMA.

[27] Saika T, Tsushima T, Nasu Y, Miyaji Y, Saegusa M, Takeda K (2010). Two instillations of epirubicin as prophylaxis for recurrence after transurethral resection of Ta and T1 transitional cell bladder cancer: a prospective, randomized controlled study. World J Urol.

[28] Solsona E, Iborra I, Ricós JV, Monrós JL, Casanova J, Dumont R (1999). Effectiveness of a single immediate mitomycin C instillation in patients with low risk superficial bladder cancer: short and long-term follow up. J Urol.

[29] Li NC, Ye ZQ, Na YQ, CUA THP® Immediate Instillations Study Group (2013). Efficacy of immediate instillation combined with regular instillations of pirarubicinf or Ta and T1 transitional cell bladder cancer after transurethral resection: a prospective, randomized, multicenter study. Chin Med J (Engl).

[30] Ueda T, Naito S, Iguchi A, Sagiyama K, Osada Y, Ariyoshi A (1992). Adjuvant chemotherapy with early intravesical instillation of adriamycin and long-term oral administration of 5-fluorouracil in superficial bladder cancer. The Kyushu University Urological Oncology Group. Cancer Chemother Pharmacol.

[31] Shen J, Chen JH, Yu QW, Shen J, Sun P (2011). Effect on prophase intravesical instillation of pirarubicin for preventing recurrence of bladder cancer. World Clin Durgs.

[32] Chen YL, Ye LH, Tao SX, Qian SX (2003). The therapeutic effects of intravesical instillation of mitomycin C immediately after TURBT for preventing recurrence of superficial bladder cancer. Zhejiang Clin Med J.

[33] Li B, Lin Z, Li J, Qu Y (2013). Comparison of THP high dose immediate perfusion and routine perfusion. Contemp Med.

[34] Li J (2017). Clinical study of immediate postoperative perfusion combined with distilled water irrigation for non-invasive urothelial carcinoma of bladder. World Latest Med Inform..

[35] Li XD, Zhao EY, Wang CL, Xu WH (2011). Clinical application of localization diagnosis and treatment in the early non-muscle invasive bladder cancer with THP. Prog Mod Biomed..

[36] Wang GQ (2013). Intravesical instillation of pirarubicin aftre transurethral resection of bladder for superficial bladder cancer. J Clin Res.

[37] Wang JG, Zhang XJ, Chen SX, Yang GH, Ding SP, Zhou JJ (2012). A therapeutic effect analysis of the comparative early intravesical instillation of pirarubicin versus traditional intravesical instillation of pirarubicin for superficial bladder cancer. J Clin Urol.

[38] Wang WM, Song YJ, Wang SC (2012). Effect of early postoperative intravesical instillation of Pirarubicin on prevention of postoperative recurrence of bladder cancer. Henan J Surg.

[39] Xu N, Xue XY, Zhou HL, Mao HP, Zheng QS, Wei Y (2008). Intravesical instillation of mitomycin C at different time after transurethral resection for the prophylaxis of superficial bladder cancer recurrence. Int J Urol Nephrol.

[40] Yu J, Gong DX, Jiang YJ (2011). The action on the recurrence of non-muscle-invasive bladder carcinoma using immediate and delayed instillation by hydroxycamptothecin. J Chin Med Univ..

[41] Zhang HY, Song XW, Tian BY, Zhang GW, Guo RM, Li DC (2010). Comparison of two different perfusion methods after resection of bladder tumor. Her Med.

[42] Zhang HF (2017). Clinical observation of postoperative bladder perfusion with pirarubicin for transurethral resection of superficial bladder cancer. Henan Med Res.

[43] Zhang JC, Li J, Zhu F, Zhang YJ (2010). Intravesical instillation of THP immediately and regularly after transurethral resection of superficial bladder cancer. China Med.

[44] Zhang YX, Tian K, Wang YL, Liu QZ, Yao DQ, Guo GF (2013). Clinical significance of instantly intravesical instillation of pirarubicin for preventing recurrence of superficial bladder cancer after transurethral resection of bladder tumor. Chin J Pract Med.

[45] Gu AZ, Zhu Q, Hu YS (2020). Clinical effect of gemcitabine combined with pirarubicin intravesical chemotherapy for low-risk bladder cancer patients after operation. Chin J Pract Med.

[46] Wu B, Wang XG, Shi HL, Mo NX, Lv Z (2017). Clinical effect of gemcitabine intravesical instillation after resection of non-muscle invasive bladder cancer and its effect on immune function. Med J Commun.

[47] Jiang BH, Cao QF, Feng QX (2019). Clinical observation of gemcitabine irrigation after transurethral resection of non-muscle invasive bladder tumor in gerontal patients. J Pract Med..

[48] Wei C (2017). Effect of intravesical instillation of gemcitabine on prevention of postoperative recurrence in patients with non muscle invasive bladder cancer. Med Front.

[49] Xiao CS (2019). Effect of intravesical instillation of gemcitabine on prevention of postoperative recurrence of bladder cancer without muscle infiltration. Strait Pharm J.

[50] Wei C, Yu Y, Ni LL, Yang HS, Yang YH (2021). Clinical efficacy of transurethral plasmakinetic resection combined with gemcitabine bladder perfusion therapy in treatment of non muscle invasive bladder cancer. Chin Foreign Med Res.

[51] Liu DC, Shi HB, Chen B (2018). A comparative study of intravesical instillation of gemcitabine and pirarubicin in the treatment of bladder cancer. Pract J Cancer.

[52] Zhang GP, Lei G, Wang L, Zhang TD, Mu JJ, Bai B (2018). Efficacy and safety of gemcitabine intravesical instillation in the treatment of non-myometrial invasive bladder cancer. Sichuan Medl J.

[53] Huang J, Xiong JY (2018). Clinical study of gemcitabine used for intravesical instillation. Chin Mod Dr.

[54] Sun J, Sun CW, Ding XQ (2018). Observation of recurrence and survival of superficial bladder cancer treated by plasma electrocision combined with gemcitabine intravesical instillation. Guizhou Med J.

[55] Zhang JJ, Cai WQ, Zhang SQ, Fang XL (2016). Comparison of efficacy between intra-bladder perfusion with gemcitabine and pirarubicin for preventing postoperative recurrence of non-muscle invasive bladder cancer. Guangxi Med J.

[56] Zhang JJ, Hao L, Shi ZY, Pang K, Dong Y, Han CH (2017). Intravesical instillation of gemcitabine and pirarubicin for prevention of postoperative recurrence of non muscle invasive bladder cancer. J Clin Med Lit (Electron Ed).

[57] Qin KS, Li JQ (2019). Effect of intravesical instillation chemotherapy with gemcitabine on transurethral resection of bladder cancer. Mod Diagn Treat.

[58] Li LJ, Ma ZW, Gong BS, Qiu MX (2016). Application of gemcitabine in high risk non muscle invasive bladder cancer after two transurethral resection. Mod Med Health.

[59] Kong LQ, Yu QC, Yang M, Qu YL (2016). Effect of gemcitabine and pirarubicin infusion on recurrence after bladder cancer surgery. Med J Qilu.

[60] Wang LC (2016). Effect of intravesical instillation chemotherapy with gemcitabine on patients with bladder cancer undergoing transurethral resection of the tumor. Contemp Med Forum.

[61] Hu P, Li G (2019). Comparison of gemcitabine and pirarubicin instillation of bladder for bladder cancer. J Clin Ration Drug Use..

[62] Cai Q, Wang ZS, Zhang W, Cao JJ (2019). Comparison of gemcitabine and pirarubicin after single time intravesical instillation chemotherapy in the treatment of low risk bladder cancer. Zhejiang J Integr Tradit Chin West Med.

[63] Dong SF (2016). Effect of pirarubicin and gemcitabine intravesical chemotherapy on the recurrence and postoperative survival of bladder cancer patients. Chin J Clin Oncol Rehabil.

[64] Feng SQ, Fan HT, Ren M, Zhang M (2014). Clinical research of postoperative intravesical instillation of gemcitabine to prevent recurrence of bladder cancer (Report of 198 cases). J Clin Urol.

[65] Jiang T, Wang DG, Jiang B, Yang Y, Guo J, Zhang JR (2016). The clinical study of the safety and tolerance of using gemcitabine in bladder perfusion Immediately after transurethral resection of bladder tumor. Med Philos.

[66] Xu T, Li JT, Yang DJ, Su YD, Li JJ (2016). Study of gemcitabine intravesical chemotherapy. Pract Pharm Clin Rem.

[67] Cheng WB, Zhang N, Li Q (2020). The value of gemcitabine perfusion in elderly patients with non-myometrial invasive bladder tumor after transurethral resection. Clin Res.

[68] Zhang XH, Tan J, Yao K, Wang JR, Wan B, Wei WS (2015). Clinical effect of anticancer drug perfusion in preventing postoperative recurrence of superficial bladder cancer. J Chin Phys.

[69] Liu XY, Xiang CH, Chen SL (2018). Curative efficacy of intravesical reperfusion with gemcitabine for preventing postoperative re-currence of non-muscle invasive bladder cancer. Pract J Clin Med.

[70] Zhang X, An RH, Yu JS, Yu SL, Gan XG (2015). Pirarubicin preventive efficacy of superficial bladder cancer recurrence. Prog Mod Biomed..

[71] Dong XC, Song LD, Zhang F (2017). Effect of intravesical infusion of different chemotherapeutic drugs on preventing tumor recurrence after TURBT. Zhejiang J Traum Surg.

[72] He YF, Ma YT, Gao WJ (2018). Efficacy and safety of intravesical chemotherapy with gemcitabine in preventing postoperative recurrence of non muscle invasive bladder cancer. Clin Res Pract..

[73] Hu YQ, Wang XR, Xie MM, Zhao Y, Wang ZJ, Xu ML (2015). The efficacy of intravesical chemotherapy for bladder cancer treat with pirarubicin and gemcitabine. J Mod Oncol.

[74] Liao YX, Zhou JJ, Yang GH, Zeng JM (2017). Study on gemcitabine in the treatment of bladder cancer. J Contemp Urol Reprod Oncol.

[75] Qiang YC, Li J (2021). Efficacy and safety of hyperthermic bladder chemotherapy with gemcitabine after transurethral resection of bladder tumors in elderly with superficial bladder cancer. Shaanxi Med J.

[76] Chen DP, Zhang Y (2008). Comparison of the therapeutic effect of three drugs by bladder instillation after transurethral resection of superficial bladder cancer. J Mod Urol.

[77] Tu FZ, Shen ZJ, Wang XJ, Zhong S, Zhu ZW, Wang HF (2012). The clinical investigation of different intravesical instillation of chemotherapy after transurethral resection of bladder tumors for the treatment of superficial bladder cancer. Int J Urol Nephrol.

[78] Guan H, Li QW (2015). Therapeutic comparison of bladder instillation with hydroxycamptothecine and pirarubicin on preventing the postoperative recurrence of superficial bladder cancer. Chin J Anat Clin.

[79] Tao JH, Jiang X (2015). Efficacy of pirarubicin combined with hydroxycamptothecin in preventing recurrence after resection of bladder cancer. Lab Med Clin.

[80] Long J, Lin HS, Mo ZN, Wei SC (2017). Clinical effects of different intravesical instillation chemotherapies on non-muscle invasive bladder cancer after transurethral electroresection. Chin J New Clin Med.

[81] Pu J, Wu XH, Tang W, Chen ZX, Wang DL, He YF (2009). Clinical study of intravesical pirarubicin instillation for preventing postoperative recurrence of superficial bladder cancer. Chongqing Med J.

[82] Wang P. Clinical observation and analysis of intravesical instillation of three drugs to prevent postoperative recurrence of superficial bladder tumor. Qinghai University; 2014.

[83] Mao WD, Wu JM, Zhang BZ, Zheng DY, Zhou JH, Xu YC (2012). Clinical efficacy of intravesical instillation of pirarubicin combined with Hydroxycamptothecin in the prevention and treatment of bladder cancer recurrence. Chin High Med Educ.

[84] He XL, Qiang YY, Zhang BB, Song HX, Jin YS (2017). Clinical effect of the pirarubicin in the treatment of non-muscle invasive bladder cancer. Int J Urol Nephrol.

[85] Feng X. Efficacy of TURBT combined with hydroxycamptothecin and pirarubicin intravesical perfusion in the treatment of SBC and its relationship with clinical characteristics. Jinan University; 2013.

[86] Li YX, Zhang KZ, Tang ZZ, Wu AM, Chi YY (2013). THP and hydroxycamptothecin for prevention of postoperative superficial bladder cancer recurrence clinical analysis. Jilin Med J.

[87] Wang YH (2014). Comparison of therapeutic effects of different perfusion drugs after electroresection of superficial bladder tumors. Med Inf.

[88] Nan Y (2014). Comparison of efficacy of intravesical instillation of hydroxycamptothecin and pirarubicin in preventing recurrence of non muscle invasive bladder cancer. Hainan Med J.

[89] Lou B, Song XX, Guo XD (2011). Observation of recurrence prevention effect of pirarubicin after bladder cancer electrocision. Med Recapitul.

[90] Chen BK, Gao LP, Yu HY, Jiang WB, Wu SJ, Lv ZW (2010). Comparison the effect and safety of pirarubicin and mitomycin by bladder instillation for preventing postoperative recurrence on the superficial bladder cancer. Chin J Clin Pharm.

[91] Gu DL (2021). Effect of pirarubicin infusion on superficial bladder cancer and its adverse reaction. Tianjin Pharm.

[92] Fang D. Clinical observation of bladder perfusion after transurethral resection of non muscle invasive bladder cancer. Taishan Medical University; 2018.

[93] Teng DH, Zhang YG, Wang XF, Li J, Huang GM, Ao M (2011). Clinical research on prevention of recurrence of superficial bladder cancer by intravesical instillation of pirarubicin or mitomycin C after transurethral resection of bladder tumor. J Shanxi Med Univ.

[94] Zhang HQ (2006). Clinical study of intravesical instillation of pirarubicin to prevent recurrence of superficial bladder tumor. J Chin Phys.

[95] Li JH, Zhu SY (2012). Effect of intravesical instillation of pirarubicin and mitomycin on postoperative recurrence of superficial bladder cancer. Chin J Clin Oncol Rehabil.

[96] Ni JT, Dong YX, Gao XK (2018). Comparison of effects of Mitomycin and Pirarubicin infusion chemotherapy in the treatment of non-muscle invasive bladder cancer after TURBT. Chin J Front Med Sci (Electron Version).

[97] Ma J, Fan DB, Liao K (2013). Comparison of the effect of different drugs by bladder instillation in treatment with superficial bladder cancer. Sichuan Med J.

[98] Ye LH (2012). Comparison of curative effect and nursing experience of pirarubicin and mitomycin intravesical instillation to prevent postoperative recurrence of superficial bladder cancer. Strait Pharma J.

[99] Zhang LB, Yang ZY, Feng G, Shen L, Hu XL, Yang B (2017). Clinical study of intravesical instillation of different drugs to prevent postoperative recurrence of superficial bladder tumor. J Clin Med Pract.

[100] Yang MG, Zhao XK, Wu ZP, Xiao N, Lv C (2008). Comparison of the therapeutic efficacy of benzalkonium bromide, pirarubicin, and mitomycin C in prevention of postoperative recurrence of superficial bladder cancer. Tumor.

[101] Yang NG, Wang SP, Wang J, Zhang J (2011). Efficacy of pirarubicin and mitomycin infusion chemotherapy in the prevention of bladder tumor recurrence. Chin Mod Med.

[102] Zhuang QH (2014). Prognosis of transurethral resection of superficial bladder tumor combined with pirarubicin perfusion. Chin Health Ind.

[103] Yin SS (2016). Effect of transurethral resection of bladder tumor combined with intravesical instillation of Pirarubicin on postoperative recurrence rate and quality of life in patients with superficial bladder cancer. Contemp Med.

[104] Zhu SL, Yang F (2013). Comparation of the effect of bladder instillation with mitomycin C and pirarubicin on preventing the postoperative recurrence of superficial bladder cancer. J Bengbu Med Coll.

[105] Yang T, Li Y, Zhang PJ, Liu JJ, Sun FZ, Wang G (2015). Comparative study of intravesical instillation of pirarubicin and mitomycin C in the prevention of recurrence of superficial bladder urothelial carcinoma after transurethral resection of bladder tumor. Mod J Integr Tradit Chin West Med.

[106] Wang T (2015). Bi WH Clincal study on intravesical instillation of priarubicin (THP) for preventing postoperative recurrence of superficial bladder cancer. J Mod Oncol.

[107] Zheng XB, Zhan ZS (2018). Clinical observation of pirarubicin infusion chemotherapy after TURBT for non muscle invasive bladder cancer. Shenzhen J Integr Tradit Chin West.

[108] Fu Y (2016). Transurethral resection of bladder tumor combined with intravesical instillation of pirarubicin in the treatment of superficial bladder cancer. Clin Res Pract.

[109] Su ZQ (2014). Comparative study of the efficacy in preventing the recurrence of superficial bladder cancer between intravesical pirarubicin(THP) and mitomycin(CMMC). Hainan Med J..

[110] Kuroda M, Niijima T, Kotake T, Akaza H, Hinotsu S (2004). Effect of prophylactic treatment with intravesical epirubicin on recurrence of superficial bladder cancer: the 6th Trial of the Japanese Urological Cancer Research Group (JUCRG): a randomized trial of intravesical epirubicin at dose of 20 mg/40 ml, 30 mg/40 ml, 40 mg/40 ml. Eur Urol.

[111] Liu B, Wang Z, Chen B, Yu J, Zhang P, Ding Q (2006). Randomized study of single instillation of epirubicin for superficial bladder carcinoma: long-term clinical outcomes. Cancer Invest.

[112] Masters JR, Popert RJ, Thompson PM, Gibson D, Coptcoat MJ, Parmar MK (1999). Intravesical chemotherapy with epirubicin: a dose response study. J Urol.

[113] Liu BC, Zhang YF, Wang Z, Ding Q, Chen B, Yu J (2004). Single intravesical instillation of epirubicin for primary superficial bladder carcinoma: long-term results. Chin J Urol.

[114] Lv J, Wang JW (2015). Cost-effectiveness analysis of epirubicin bladder perfusion in preventing postoperative recurrence of superficial bladder cancer. Chin Pharm.

[115] Liang JM, Yang ZB, Wang XM, Mo LJ, Yu J (2015). The clinical research on preventing the recurrence of superficial bladder cancer by intravesical infusion with epirubicin and mitomycin. J Guangxi Med Univ.

[116] Zhang YF, Zhang ZB, Tang YZ, Liu BC, Sun FK, Tang H (2000). Single or consecutive instillation of epirubicin or mitomycin C for the prophylaxis of recurrent primary superficial bladder carcinoma. Chin Oncol.

[117] Wu ZB, Lin GB, Chen BJ, Wu ZM, Rong RM (2005). Efficacy and safty of different dosages of intravesical epirubicin instillation for prevention of primary superficial bladder carcinoma from recurrence. Chin J Oncol.

[118] Deng XJ, Li ZM, Li S, Li G, Huang WS, Shi GQ (2017). Effectiveness and safety of different doses of pirarubicin on preventing non-muscle invasive bladder cancer recurrence by intravesical instillation. J Clin Urol.

[119] Peng J, Pan M (2011). Single bladder pirarubicin perfusion to prevent the recurrence of superficial bladder carcinoma. J Contemp Urol Reprod Oncol.

[120] Lu JL, Xia QD, Lu YH, Liu Z, Zhou P, Hu HL (2020). Efficacy of intravesical therapies on the prevention of recurrence and progression of non-muscle-invasive bladder cancer: a systematic review and network meta-analysis. Cancer Med.

[121] De Reijke TM, Kurth KH, Sylvester RJ, Hall RR, Brausi M, Van De Beek K (2005). Bacillus Calmette-Guerin versus epirubicin for primary. Secondary or concurrent carcinoma in situ of the bladder: results of a european organization for the research and treatment of Cancer-Genito-Urinary Group phase III trial (30906). J Urol.

[122] Di Lorenzo G, Perdonà S, Damiano R, Faiella A, Cantiello F, Pignata S (2010). Gemcitabine versus bacille Calmette–Guérin after initial bacille Calmette–Guérin failure in non-muscle-invasive bladder cancer: a multicenter prospective randomized trial. Cancer.

[123] Fallah F, Fallah M, Sajadi Nia RS (2012). Thiotepa versus bacille Calmette–Guérin in non-muscle invasive bladder cancer. Current Urol.

[124] Gontero P, Oderda M, Mehnert A, Gurioli A, Marson F, Lucca I (2013). The impact of intravesical gemcitabine and 1/3 dose bacillus Calmette-Guerin instillation therapy on the quality of life in patients with nonmuscle invasive bladder cancer: results of a prospective, randomized, phase II trial. J Urol.

[125] Hinotsu S, Akaza H, Naito S, Ozono S, Sumiyoshi Y, Noguchi S (2011). Maintenance therapy with bacillus Calmette-Guerin Connaught strain clearly prolongs recurrence-free survival following transurethral resection of bladder tumour for non-muscle-invasive bladder cancer. BJU Int.

[126] Lamm DL, Blumenstein BA, Crawford ED, Montie JE, Scardino P, Grossman HB (1991). A randomized trial of intravesical doxorubicin and immunotherapy with bacille Calmette-Guérin for transitional-cell carcinoma of the bladder. N Engl J Med.

[127] Lamm DL, Blumenstein BA, David Crawford E, Crissman JD, Lowe BA, Smith JA (1995). Randomized intergroup comparison of bacillus calmette-guerin immunotherapy and mitomycin C chemotherapy prophylaxis in superficial transitional cell carcinoma of the bladder a southwest oncology group study. Urol Oncol.

[128] Lundholm C, Norlén BJ, Ekman P, Jahnson S, Lagerkvist M, Lindeborg T (1996). A randomized prospective study comparing long-term intravesical instillations of mitomycin C and bacillus Calmette-Guerin in patients with superficial bladder carcinoma. J Urol.

[129] Martínez-Piñeiro JA, Jiménez León J, Martínez-Piñeiro L, Fiter L, Mosteiro JA, Navarro J (1990). Bacillus Calmette–Guerin versus doxorubicin versus thiotepa: a randomized prospective study in 202 patients with superficial bladder cancer. J Urol.

[130] Melekos MD, Chionis HS, Paranychianakis GS, Dauaher HH (1993). Intravesical 4'-epi-doxorubicin (epirubicin) versus bacillus Calmette-Guérin. A controlled prospective study on the prophylaxis of superficial bladder cancer. Cancer.

[131] Rintala E, Jauhiainen K, Alfthan O, Hansson E, Juusela H, Kanerva K (1991). Intravesical chemotherapy (mitomycin C) versus immunotherapy (bacillus Calmette-Guérin) in superficial bladder cancer. Eur Urol.

[132] van der Meijden AP, Brausi M, Zambon V, Kirkels W, de Balincourt C, Sylvester R (2001). Intravesical instillation of epirubicin, bacillus Calmette-Guerin and bacillus Calmette-Guerin plus isoniazid for intermediate and high risk Ta, T1 papillary carcinoma of the bladder: a European Organization for Research and Treatment of Cancer genito-urinary group randomized phase III trial. J Urol.

[133] Vegt PD, Witjes JA, Witjes WP, Doesburg WH, Debruyne FM, van der Meijden AP (1995). A randomized study of intravesical mitomycin C, bacillus Calmette-Guerin Tice and bacillus Calmette-Guerin RIVM treatment in pTa-pT1 papillary carcinoma and carcinoma in situ of the bladder. J Urol.

[134] Witjes JA, van der Meijden AP, Witjes WP, Doesburg W, Schaafsma HE, Debruyne FM (1993). A randomised prospective study comparing intravesical instillations of mitomycin-C, BCG-Tice, and BCG-RIVM in pTa-pT1 tumours and primary carcinoma in situ of the urinary bladder. Eur J Cancer.

[135] Li JX, Li JB, Zhang P, Lin SQ, Sun Y (2009). Intravesical instillation of piarubicin for preventing postoperative recurrence of superficial bladder cancer. J Trop Med.

[136] Xia JP, Deng LH, Xu XH, Tang CL (2011). Clinical observation of preventing bladder cancer postoperative recurrence by BCG intravesical instillation combined with pirarubicin. Chin J Med Guide.

[137] Pei L (2015). Observation on the efficiency of different drugs by bladder instillation for prevention of recurrences in patients with superficial bladder carcinoma. Shanxi Med J.

[138] Wang S, Yu JQ, Xia D, Shen BH, Jin BY, Chen SY (2003). Single-dose instillations of epirubicin as prophylaxis for recurrence of superficial bladder tumors: a prospective randomized controlled study. Chin J Urol.

[139] Yu SY (2011). Efficacy of mitoxantrone hydrochloride and BCG intravesical infusion in preventing tumor recurrence. Pract Pharm Clin Rem.

[140] Yang WX, Shao YX, Hu X, Xiong SC, Liu Y, Li X (2021). A randomized controlled study of intravesical instillation therapy of bacillus Calmette-Guérin vs. Epirubicinin treating non-muscular invasive bladder cancer. J Sichuan Univ (Med Sci Edition)..

[141] Ma WJ, Shao T, Shuang WB, Wang DW (2009). A randomized controlled study of single intravesical instillation of pirarubicin in the prevention of postoperative recurrence of non myometrial invasive bladder urothelial carcinoma. Cancer Res Clin.

[142] Wu XD, Yang XZ, Wang J (2006). Observarion on the effect of THP, BCG by bladder instillation to prevent the recurrence of the bladder cancer. Int Med Health Guid News.

[143] Ning YC (2013). Comparison of two different intravesical chemotherapy efficacy in the prevention of superficial bladder cancer recurrence. World Latest Med Inf.

[144] Kang YS, Sun DN, Wang Q, Yan JQ, Meng MS, Sun DD (2009). Comparison between pirarubicin and BCG bladder instillation for prevention of superficial bladder cancer from recurrence. Med J Natl Defend Forces Southwest Chin.

[145] Shi ZY, Zhou SY, Yi HP (2001). Comparison of the therapeutic effect of several drugs infused into the bladder for superficial tumors after transurethral resection of the bladder tumor. Chin Oncol.

[146] Schmidt S, Kunath F, Coles B, Draeger DL, Krabbe LM, Dersch R (2020). Intravesical Bacillus Calmette-Guérin versus mitomycin C for Ta and T1 bladder cancer. Cochrane Database Syst Rev.

[147] Li XY, Cai Y, Hu X, Wang YB, Wang YY, Jin YH (2021). Efficacy and safety of low dose and standard dose bacillus calmette guerin vaccine for non-muscle invasive bladder cancer: a meta-analysis. J Contemp Urol Reprod Oncol..

[148] Oddens J, Brausi M, Sylvester R, Bono A, Van De Beek C, Van Andel G (2013). Final results of an EORTC-GU cancers group randomized study of maintenance bacillus Calmette-Guérin in intermediate- and high-risk Ta, T1 papillary carcinoma of the urinary bladder: one-third dose versus full dose and 1 year versus 3 years of maintenance. Eur Urol.

[149] Qin DM, Yang JR, Li XH, Lou JA, Wang YY, Wang YB (2021). Efficacy of bacille Calmette-Guérin induction therapy and bacille Calmette-Guérin induction plus with maintenance therapy for postoperative non-muscle invasive bladder cancer: a systematic review and Meta-analysis. Chin J Front Med Sci (Electron Version)..

[150] Kamat AM, Lerner SP, O’donnell M, Georgieva MV, Yang M, Inman BA (2020). Evidence-based assessment of current and emerging bladder-sparing therapies for non-muscle-invasive bladder cancer after Bacillus Calmette-Guerin therapy: a systematic review and meta-analysis. Eur Urol Oncol..

[151] Li R, Sundi D, Zhang J, Kim Y, Sylvester RJ, Spiess PE (2020). Systematic review of the therapeutic efficacy of bladder-preserving treatments for non-muscle-invasive bladder cancer following intravesical Bacillus Calmette-Guérin. Eur Urol.

[152] Huang D, Jin YH, Weng H, Huang Q, Zeng XT, Wang XH (2019). Combination of intravesical Bacille Calmette-Guérin and chemotherapy vs. Bacille Calmette-Guérin alone in non-muscle invasive bladder cancer: a meta-analysis. Front Oncol.

[153] Arends TJ, Nativ O, Maffezzini M, De Cobelli O, Canepa G, Verweij F (2016). Results of a randomised controlled trial comparing intravesical chemohyperthermia with mitomycin c versus bacillus Calmette-Guérin for adjuvant treatment of patients with intermediate- and high-risk non-muscle-invasive bladder cancer. Eur Urol.

[154] Di Stasi SM, Giannantoni A, Stephen RL, Capelli G, Navarra P, Massoud R (2003). Intravesical electromotive mitomycin C versus passive transport mitomycin C for high risk superficial bladder cancer: a prospective randomized study. J Urol.

[155] Droller D (2002). Equivalent efficacy of mitomycin C plus doxorubicin instillation to bacillus Calmette-Guerin therapy for carcinoma in situ of the bladder. J Urol.

[156] van Gils-Gielen RJ, Witjes WP, Caris CT, Debruyne FM, Witjes JA, Oosterhof GO (1995). Risk factors in carcinoma in situ of the urinary bladder. Dutch South East Cooperative Urological Group. Urology.

[157] Witjes JA, van Meijden AP, Collette L, Sylvester R, Debruyne FM, Van Aubel A (1998). Long-term follow-up of an EORTC randomized prospective trial comparing intravesical bacille Calmette-Guérin-RIVM and mitomycin C in superficial bladder cancer. EORTC GU Group and the Dutch South East Cooperative Urological Group. European Organisation for Research and Treatment of Cancer Genito-Urinary Tract Cancer Collaborative Group. Urology.

