# Supplementary material for: Treatment and surveillance for non-muscle-invasive bladder cancer: a clinical practice guideline (2021 edition)
Source: Mil Med Res. 2022 Aug 17;9:44. doi: 10.1186/s40779-022-00406-y (PMC9382792; doi:10.1186/s40779-022-00406-y)
Supplement: Supplementary file 1 — Additional file 1. Classification criterion of NMIBC [file 40779_2022_406_MOESM1_ESM.pdf]

Table S1 2017 TNM classification [1]

Stage	Criterion
**T (Primary tumor)**	
Tx	Primary tumor cannot be assessed
T0	No evidence of primary tumor
Ta	Non-invasive papillary carcinoma
Tis	Carcinoma in situ: ‘flat tumor’
T1	Tumor invades subepithelial connective tissue
T2	Tumor invades muscle
T2a	Tumor invades superficial muscle (inner half)
T2b	Tumor invades deep muscle (outer half)
T3	Tumor invades perivesical tissue
T3a	Microscopically
T3b	Macroscopically (extravesical mass)
T4	Tumor invades any of the following: prostate stroma, seminal vesicles, uterus, vagina, pelvic wall, abdominal wall
T4a	Tumor invades prostate stroma, seminal vesicles, uterus or vagina
T4b	Tumor invades pelvic wall or abdominal wall
**N (Regional lymph nodes)**	
Nx	Regional lymph nodes cannot be assessed
N0	No regional lymph node metastasis
N1	Metastasis in a single lymph node in the true pelvis (hypogastric, obturator, external iliac, or presacral)
N2	Metastasis in multiple regional lymph nodes in the true pelvis (hypogastric, obturator, external iliac, or presacral)
N3	Metastasis in common iliac lymph node (s)
**M (Distant metastasis)**	
M0	No distant metastasis
M1a	Non-regional lymph nodes
M1b	Other distant metastases

TNM Tumor Node Metastasis

Table S2 WHO classifications in 1973 and in 2004/2016 [2-3]

2004/2016 WHO classification system (papillary lesions)	WHO 1973 classification system
Papillary urothelial neoplasm of low malignant potential (PUNLMP)	Grade 1: well differentiated
Low-grade (LG) papillary urothelial carcinoma	Grade 2: moderately differentiated
High-grade (HG) papillary urothelial carcinoma	Grade 3: poorly differentiated

WHO World Health Organization

Table S3 Risk group definition used in the guideline [4]

Risk group	Criterion
Low risk	Primary, solitary, TaG1 (PUNLMP, low grade), tumor diameter < 3 cm, no CIS
Intermediate risk	All tumors not defined in the two adjacent categories (between the category of low- and high-risk)
High risk	Any of the following:
	(1) T1 tumor
	(2) G3 (high grade) tumor
	(3) CIS
	(4) Multiple, recurrent and large (> 3 cm) TaG1G2/ low grade tumors (all features must be present)

PUNLMP papillary urothelial neoplasm of low malignant potential, *CIS* carcinoma in situ, tumor in situ

Table S4 Risk group definition in EAU-NMIBC guideline [4]

**Risk group**	**Criterion**
Low risk	(1) A primary, single, Ta/T1 LG/G1 tumor < 3 cm in diameter without CIS in a patient < 70 years
	(2) A primary Ta LG/G1 tumor without CIS with at most ONE of the additional clinical risk factors[*]
Intermediate risk	Patients without CIS who are not included in either the low, high or very high-risk groups
High risk	(1) All T1 HG/G3 without CIS, EXCEPT those included in the very high-risk group
	(2) All CIS patients, EXCEPT those included in the very high-risk group
	Stage, grade with additional clinical risk factors:
	(1) Ta LG/G2 or T1 G1, no CIS with all 3 risk factors[*]
	(2) Ta HG/G3 or T1 LG, no CIS with at least 2 risk factors[*]
	(3) T1 G2 no CIS with at least 1 risk factor[*]
Very high risk	Stage, grade with additional clinical risk factors:
	(1) Ta HG/G3 and CIS with all 3 risk factors[*]
	(2) T1 G2 and CIS with at least 2 risk factors[*]
	(3) T1 HG/G3 and CIS with at least 1 risk factor[*]
	(4) T1 HG/G3 no CIS with all 3 risk factors[*]

[*]Clinical risk factors: age > 70; multiple papillary tumors; tumor diameter > 3 cm. *CIS* carcinoma in situ, tumor in situ, *EAU* European Association of Urology, *HG* high grade, *LG* low grade, *NMIBC* non-muscle-invasive bladder cancer

References

1. Brierley J, Gospodarowicz M, Wittekind C. TNM classification of malignant tumors. UICC International Union Against Cancer. 8th ed. New York: Wiley-Blackwell and UICC; 2017.

2. Moch H, Humphrey PA, Ulbright TM, Reuter VE. WHO Classification of Tumours of the Urinary System and Male Genital Organs. 4th ed. https://publications.iarc.fr/Book-And-Report-Series/Who-Classification-Of-Tumours/WHO-Classification-Of-Tumours-Of-The-Urinary-System-And-Male-Genital-Organs-2016.

3. Mostofi F, Sorbin L, Torloni H. Histologic typing of urinary bladder tumours. International classification of tumours, No 10. Geneva: WHO; 1973.

4. European Association of Urology. Non-muscle-invasive Bladder Cancer. https://uroweb.org/guideline/non-muscle-invasive-bladder-cancer/.
