# Supplementary material for: Treatment and surveillance for non-muscle-invasive bladder cancer: a clinical practice guideline (2021 edition)
Source: Mil Med Res. 2022 Aug 17;9:44. doi: 10.1186/s40779-022-00406-y (PMC9382792; doi:10.1186/s40779-022-00406-y)
Supplement: Supplementary file 2 — Additional file 2. Summary of recommendations from relevant guidelines and monographs [file 40779_2022_406_MOESM2_ESM.pdf]

Question 1: What are the indications for TURBT in NMIBC patients?

(1) EAU guideline [1]: In patients suspected of having bladder cancer, perform a TURBT followed by pathology investigation of the obtained specimen(s) as a diagnostic procedure and initial treatment step. (**Recommendation strength rating: Strong**)

(2) AUA guideline [2]: At initial diagnosis of a patient with bladder cancer, a clinician should perform complete visual resection of the bladder tumor(s), when technically feasible. (**Clinical principle**)

(3) CUA guideline [3]: Patients presenting with a bladder tumor should undergo initial TURBT for diagnostic confirmation and pathological evaluation. (**LE 2; Strong recommendation**)

(4) Chinese Guidelines for Diagnosis and Treatment of Urology and Andrology [4]: TURBT is the main treatment for NMIBC. (**Recommendation strength rating: Strong**)

Question 3: What is the extent of initial TURBT resection in NMIBC patients?

(1) EAU guideline [1]: The resection depth should reach the muscle layer with the exception of Ta-LG/G1 tumors (LE: 2b). Perform en-bloc resection or resection in fractions (exophytic part of the tumor, the underlying bladder wall and the edges of the resection area). (**Recommendation strength rating: Strong**)

(2) CUA guideline [3]: Initial TURBT aims for complete tumor resection with sampling of the underlying detrusor muscle as the first step of curative-intent treatment of NMIBC (Recommendation strength rating: Strong). Patients with presumed LG Ta or CIS might be spared from muscle sampling at initial TURBT. (**LE 3, Weak recommendation**)

(3) NICE guideline [5]: Obtain detrusor muscle during TURBT.

(4) NCCN guideline [6]: Adequate resection with muscle in specimen for pathological staging. (**Recommendation category: 2a**)

(5) Guidelines for Diagnosis and Treatment of Urology and Andrology in China [4]: Resect all endoscopically visible tumors deep into the muscular layer. Tumors with a diameter of less than 1 cm could be resected with part of the underlying bladder wall for pathological diagnosis. Perform resection in fractions for large tumors until normal bladder wall is exposed. Muscle tissue should be included in the pathological specimen, and avoid tissue damage caused by cauterization as much as possible.

Question 4: What are the indications for fluorescence or narrow-band imaging guided TURBT in patients with NMIBC?

(1) EAU guideline [1]: Use methods to improve tumor visualization (fluorescence cystoscopy, narrow-band imaging) during TURBT, if available. (**Recommendation strength rating: Weak**)

(2) NICE guideline [5]: Offer white-light-guided TURBT with one of the photodynamic diagnoses, narrow-band imaging, cytology or a urinary biomarker test to people with suspected bladder cancer.

(3) Guidelines for Diagnosis and Treatment of Urology and Andrology in China [4]: Fluorescence or narrow-band imaging cystoscopy could be performed for patients with suspected CIS.

Question 5: What are the indications for secondary TURBT in patients with NMIBC?

(1) EAU guideline [1]: Second TURBT is required in the following cases: 1) after incomplete initial TURBT, or in case of doubt about completeness of a TURBT; 2) if there is no detrusor muscle in the specimen after initial resection, with the exception of Ta-LG/G1 tumors and primary CIS; 3) in T1 tumors (**Recommendation strength rating: Strong**).

(2) AUA guideline [2]: Second TURBT is required in the following cases: 1) incomplete initial TURBT (**Strong recommendation; Evidence strength: Grade B**); 2) with high-risk, HG Ta tumors (Moderate recommendation; Evidence strength: Grade C); 3) in T1 tumors (**Strong recommendation; Evidence strength: Grade B**).

(3) CUA guideline [3]: Second TURBT is required in the following cases: 1) in patients with T1 NMIBC, or when a complete resection was not achieved with the first TURBT (**Strong recommendation**); 2) in select cases of HG Ta tumors (e.g., large and/or multiple tumors) (**Weak recommendation**).

(4) NICE guideline [5]: Second TURBT is required in the following cases: 1) specimen in the first TURBT does not include detrusor muscle; 2) the first TURBT shows high-risk NMIBC.

(5) NCCN guideline [6]: Second TURBT is required in the following cases: 1) incomplete initial resection; 2) no muscle in the original specimen for HG disease; 3) large (≥ 3 cm) or multifocal lesions; 4) any T1 lesion (**Recommendation category: 2a**).

(6) Chinese Expert Consensus on Secondary Resection of Non-muscle-invasive Bladder Cancer [7]: Second TURBT is required in the following cases: 1) incomplete initial resection; 2) specimen in the first TURBT does not include muscle; 3) in T1 tumors; 4) G3 HG tumors, except for CIS.

(4) Guidelines for Diagnosis and Treatment of Urology and Andrology in China [4]: Second TURBT is required in the following cases: 1) incomplete initial resection; 2) specimen in the first TURBT does not include muscle (except for LG/G1 Ta tumors and CIS); 3) in T_1 tumors.

Question 6: How long is the recommended interval between initial and second TURBT for a NMIBC patient?

(1) EAU guideline [1]: If indicated, perform a second TURBT within 2 – 6 weeks after initial resection. This second TURB should include resection of the primary tumor site. (**Recommendation strength rating: Weak**)

(2) AUA guideline [2]: For a patient with high-risk, HG Ta tumors, performing a second TURBT with repeat transurethral resection of the primary tumor site should be considered within 6 weeks of the initial TURBT (Moderate recommendation). For a patient with T1 disease, a second TURBT should be performed within 6 weeks of the initial TURBT. (**Strong recommendation**)

(3) CUA guideline [3]: A second TURBT of the primary tumor site to include muscularis propria should be performed within 6 weeks of the initial TURBT. (**Weak recommendation**)

(4) NICE guideline [5]: Consider further TURBT within 6 weeks if the first specimen does not include detrusor muscle. If the first TURBT shows high-risk NMIBC, offer another TURBT as soon as possible and no later than 6 weeks after the first resection.

(5) NCCN guideline [6]: Conduct a second TURBT within 6 weeks after the initial resection. (**Recommendation category: 2a**)

(6) Chinese Expert Consensus on Secondary Resection of Non-muscle-invasive Bladder Cancer [7]: Conducting a second TURBT within 6 weeks after the initial resection is recommended.

(7) Guidelines for Diagnosis and Treatment of Urology and Andrology in China [4]: Second TURBT within 2 – 6 weeks after the initial resection is suggested.

Question 7: What are the contraindications of immediate postoperative intravesical chemotherapy?

(1) EAU guideline [1]: Omit a single immediate instillation of chemotherapy in any case of overt or suspected bladder perforation or bleeding. (**Recommendation strength rating: Strong**)

(2) AUA guideline [2]: Do not use postoperative intravesical chemotherapy in a patient with a suspected perforation or extensive resection. (**Moderate recommendation; Evidence strength: Grade B**)

(3) CUA guideline [3]: Single instillation of chemotherapy (SIC) should not be administered after extensive resection or when bladder perforation is suspected. (**LE 3, Strong recommendation**)

(4) NCCN guideline [6]: An immediate post-operative intravesical chemotherapy is contraindicated in patients with bladder perforation and known drug allergy. (**Recommendation category: 2a**)

(5) Guidelines for Diagnosis and Treatment of Urology and Andrology in China [4]: An immediate post-operative intravesical chemotherapy is not recommended when there is TURBT intra-operative bladder perforation or severe postoperative gross hematuria.

Question 8: Does immediate postoperative intravesical chemotherapy reduce the risk of recurrence of NMIBC?

(1) EAU guideline [1]: Single instillation might have an impact on recurrence even when further adjuvant chemotherapy instillations are given. (LE: 3). If intravesical chemotherapy is given, administer a single immediate instillation of chemotherapy within 24 h after TURBT. (**Recommendation strength rating: Weak**)

(2) AUA guideline [2]: In a patient with suspected or known low- or intermediate-risk bladder cancer, a single postoperative instillation of intravesical chemotherapy (e.g., gemcitabine, mitomycin-C) should be administered within 24 h of TURBT. (**Moderate recommendation; Evidence strength: Grade B**)

(3) CUA guideline [3]: SIC (with mitomycin-C, epirubicin, doxorubicin, pirarubicin, or gemcitabine) should be offered to all patients with presumed low-risk NMIBC at TURBT and should be administered within 24 h after endoscopic resection (LE 1, strong recommendation). SIC is recommended for intermediate-risk NMIBC and for patients with ≤ 1 recurrence/year and European Organization for Research and Treatment of Cancer (EORTC) recurrence score < 5. (**LE 1; strong recommendation**)

(4) NICE guideline [5]: Offer people with suspected bladder cancer a single dose of intravesical mitomycin-C given at the same time as the first TURBT.

(5) NCCN guideline [6]: A single instillation of chemotherapy is administered within 24 h of surgery (ideally within 6 h) (**Recommendation category: 2a**). Immediate

postoperative intravesical chemotherapy reduces the 5-year recurrence rate by approximately 35% (**Recommendation category: 2a**). Immediate postoperative intravesical chemotherapy is not effective in patients with an elevated EORTC recurrence risk score (≥5) (**Recommendation category: 2a**).

(6) Guidelines for Diagnosis and Treatment of Urology and Andrology in China [4]: Immediate intravesical chemotherapy after TURBT significantly reduces the recurrence rate of NMIBC.

Question 9: Do patients with low-risk tumors only need SIC?

(1) EAU guideline [1]: In low-risk patients, a single instillation reduces the risk of recurrence and is considered to be the standard and complete treatment (**LE: 1a**); For other patients, however, a single instillation remains an incomplete treatment because of the considerable likelihood of recurrence and/or progression.

(2) AUA guideline [2]: Induction intravesical therapy should not be administered in a low-risk patient (**Moderate recommendation; Evidence strength: Grade C**).

(3) CUA guideline [3]: No further treatment is needed other than a single postoperative instillation of intravesical chemotherapy following TURBT for low-risk patients.

(4) NICE guideline [5]: Offer people with suspected bladder cancer a single dose of intravesical mitomycin-C given at the same time as the first TURBT.

(5) NCCN guideline [6]: Adjuvant therapy depends on the probability of recurrence and progression, SIC after TURBT is complete for patients with low recurrence risk.

(6) Guidelines for Diagnosis and Treatment of Urology and Andrology in China [4]: After immediate postoperative intravesical chemotherapy in low-risk NMIBC patients, maintenance intravesical chemotherapy is not recommended.

Summary of recommendations on chemotherapy for NMIBC patients with different risk levels referring to previous guidelines and monographs are shown in **Table S1**.

Table S1 Summary of recommendations on chemotherapy for NMIBC patients referring to relative guidelines and monographs

Guidelines/ monographs	Risk level		
	**Low**	**Intermediate**	**High**
EAU guideline [1]	SIC	SIC + full-dose intravesical BCG immunotherapy (induction plus 3-weekly instillations at 3, 6 and 12 months); or SIC+ instillations of chemotherapy (less than 1 year)	SIC + full-dose intravesical BCG immunotherapy for 1 to 3 years (induction plus 3-weekly instillations at 3, 6, 12, 18, 24, 30 and 36 months)
AUA guideline [2]	SIC	SIC + 6-week course of induction chemotherapy+ maintenance chemotherapy (after complete response to the induction therapy); or SIC + 6-week course of induction BCG +maintenance BCG for 1 year (after complete response to the induction therapy)	SIC + 6-week course of induction BCG + maintenance chemotherapy for 3 years or maintenance BCG (after complete response to the induction therapy)
CUA guideline [3]	SIC	SIC + intravesical induction chemotherapy + subsequent monthly maintenance for up to 1 year; or SIC+ induction BCG + maintenance BCG	SIC+ induction BCG (weekly instillations for 6 weeks) + 3-year maintenance (weekly instillations for three weeks at 3, 6, 12, 18, 24, 30, and 36 months)
NICE guideline [5]	A single dose of intravesical mitomycin-C	A single dose of intravesical mitomycin-C + a course of at least 6 doses of intravesical mitomycin-C	A single dose of intravesical mitomycin-C + intravesical BCG or RC
Guidelines for Diagnosis and Treatment of Urology and	SIC	SIC+ induction instillations + maintenance instillations; induction instillations: once a week for 4 to 8 weeks after surgery; maintenance instillations: monthly maintenance for 6 to 12 months	

Andrology in China [4]

EAU European Association of Urology, *SIC* Single postoperative instillation of intravesical chemotherapy, *BCG* Bacillus Calmette-Guerin, *AUA* American Urological Association, *CUA* Canadian Association of Urology, *NICE* The National Institute for Health and Care Excellence, *RC* Radical cystectomy

Question 10: What are the commonly used drugs and doses of intravesical chemotherapy?

(1) EAU guideline [1]: The optimal schedule and duration of further intravesical chemotherapy instillation is not defined. (**Recommendation strength rating: weak**)

(2) NICE guideline [5]: Offer people with suspected bladder cancer a single dose of intravesical mitomycin-C given at the same time as the first TURBT. Offer people with newly diagnosed intermediate-risk non-muscle-invasive bladder cancer a course of at least 6 doses of intravesical mitomycin-C.

(3) NCCN guideline [6]: The most commonly used agents are BCG, mitomycin-C, and gemcitabine. Preferable alternatives to BCG include mitomycin-C or gemcitabine. Other options include epirubicin, valrubicin, docetaxel, or sequential gemcitabine/docetaxel, sequential gemcitabine/mitomycin-C. (**Recommendation category: 2a**)

(4) Guidelines for Diagnosis and Treatment of Urology and Andrology in China [4]: Commonly used agents include pirubicin (normal dose: 30 – 50 mg), epirubicin (normal dose: 50 – 80 mg), doxorubicin (normal dose: 30 – 50 mg), hydroxycamptothecin (normal dose: 10 – 20 mg), mitomycin-C (normal dose: 20 – 60 mg), and gemcitabine (normal dose: 1000 g).

Question 11: How to improve the efficacy of intravesical chemotherapy?

(1) EAU guideline [1]: If intravesical chemotherapy is given, use the drug at its optimal pH and maintain the concentration of the drug by reducing fluid intake before and during instillation (Recommendation strength rating: Strong). The length of individual instillations should be 1 – 2 h. (**Recommendation strength rating: weak**)

(2) Guidelines for Diagnosis and Treatment of Urology and Andrology in China [4]: The efficacy of intravesical chemotherapy depends on urine pH and drug concentration. Chemotherapeutic drugs should be instilled through urethral catheter and be left in for 0.5 – 2 h (according to the drug instructions). Avoid drinking too much water before instillation, and select appropriate solvent according to the drug instructions.

Question 13: What are the contraindications of intravesical BCG immunotherapy?

(1) EAU guideline [1]: Absolute contraindications of BCG intravesical instillation are: 1) during the first two weeks after TURB; 2) in patients with visible haematuria; 3) after traumatic catheterization; 4) in patients with symptomatic urinary tract infection. (**Recommendation strength rating: Strong**)

(2) NCCN guideline [6]: Contraindications of BCG intravesical instillation include: 1) traumatic catheterization; 2) bacteriuria; 3) persistent gross hematuria; 4) persistent severe local symptoms, or systemic symptoms (**Recommendation category: 2a**).

(3) Guidelines for Diagnosis and Treatment of Urology and Andrology in China [4]: Contraindications of BCG intravesical instillation include: 1) symptomatic urinary tract infection; 2) patients with active tuberculosis; 3) within 2 weeks after bladder surgery; 4) gross hematuria; 5) people with immune deficiency or impairment (such as AIDS patients, those undergoing immunosuppressant or radiation therapy); 6) allergic to BCG.

Question 14: Is intravesical BCG immunotherapy better than intravesical chemotherapy in patients with NMIBC?

(1) EAU guideline [1]: In patients with intermediate-risk tumors, one-year full-dose BCG treatment (induction plus 3-weekly instillations at 3, 6 and 12 months), or instillations of chemotherapy for a maximum of 1 year is recommended (**Recommendation strength rating: Strong**). In patients with high-risk tumors, full-dose intravesical BCG for 1 – 3 years (induction plus 3-weekly instillations at 3, 6, 12, 18, 24, 30 and 36 months), is indicated. (**Recommendation strength rating: Strong**)

(2) AUA guideline [2]: In intermediate-risk patients, a 6-week course of induction intravesical chemotherapy or immunotherapy can be considered (**Moderate recommendation; Evidence strength: Grade B**). Maintenance chemotherapy (**Conditional recommendation; Evidence strength: Grade C**) or Maintenance BCG (**Moderate recommendation; Evidence strength: Grade C**) is suggested for those who respond completely to the induction instillation. In high-risk patients who completely respond to induction BCG, maintenance BCG for 3 years, as tolerated, should be continued. (**Moderate recommendation; Evidence Strength: Grade B**)

(3) CUA guideline [3]: In patients with intermediate-risk NMIBC, adjuvant induction intravesical chemotherapy (**LE1; Strong recommendation**) with subsequent monthly maintenance for up to one year (**LE 3; Weak recommendation**), or induction BCG with maintenance therapy should be considered. In patients with high-risk NMIBC, BCG

therapy with induction (weekly instillations for 6 weeks) followed by 3-year maintenance (weekly instillations for three weeks at 3, 6, 12, 18, 24, 30, and 36 months) is the standard care for reducing disease recurrence and progression rates (**LE 1; Strong recommendation**).

(4) NICE guideline [5]: Offer people with intermediate-risk NMIBC a course of at least 6 doses of intravesical mitomycin-C. Offer the choice of intravesical BCG or radical cystectomy to people with high-risk NMIBC.

(5) Guidelines for Diagnosis and Treatment of Urology and Andrology in China [4]: BCG is recommended for patients with intermediate- and high-risk NMIBC as well as CIS, but not for low-risk patients.

Question 15: Is a standard dose of BCG immunotherapy superior to a low dose of BCG immunotherapy for patients with intermediate-risk and high-risk NMIBC?

(1) EAU guideline [1]: In patients with intermediate-risk tumors, one-year full- dose BCG treatment (induction plus 3-weekly instillations at 3, 6 and 12 months), or instillations of chemotherapy for a maximum of 1 year is recommended (**Recommendation strength rating: Strong**). In patients with high-risk tumors, full-dose intravesical BCG for one to 3 years (induction plus 3-weekly instillations at 3, 6, 12, 18, 24, 30 and 36 months), is indicated (**Recommendation strength rating: Strong**).

(2) NCCN guideline [6]: Dose reduction is encouraged if there are substantial local symptoms during maintenance therapy.

(3) Guidelines for Diagnosis and Treatment of Urology and Andrology in China [4]: The optimal dose of intravesical BCG therapy has not been defined.

Question 16: Is BCG induction plus maintenance instillation better than BCG induction instillation alone in patients with NMIBC?

(1) EAU guideline [1]: In patients with intermediate-risk tumors, 1-year full-dose BCG treatment (induction plus 3-weekly instillations at 3, 6 and 12 months), or instillations of chemotherapy for a maximum of 1 year is recommended (**Recommendation strength rating: Strong**). In patients with high-risk tumors, full-dose intravesical BCG for 1 – 3 years (induction plus 3-weekly instillations at 3, 6, 12, 18, 24, 30 and 36 months), is indicated (**Recommendation strength rating: Strong**).

(2) AUA guideline [2]: A 6-week course of induction intravesical chemotherapy or immunotherapy should be considered in an intermediate-risk patient (**Moderate recommendation; Evidence strength: Grade B**). Maintenance chemotherapy (**Conditional recommendation; Evidence strength: Grade C**) or BCG (**Moderate recommendation; Evidence strength: Grade C**) could be utilized for those who completely respond to the induction. In a high-risk patient with CIS, high-grade T1, or high-

risk Ta urothelial carcinoma, 6-week induction course of BCG should be administered (**Strong recommendation; Evidence strength: Grade B**). For those who completely respond to induction BCG, maintenance BCG for 3 years should be continued (**Moderate recommendation; Evidence strength: Grade B**).

(3) CUA guideline [3]: When BCG is administered for intermediate-risk NMIBC, induction (weekly instillations for 6 weeks) followed by one-year maintenance (weekly instillations for three weeks at 3, 6, and 12 months) is recommended (**LE 1; Strong recommendation**). In patients with high-risk NMIBC, BCG therapy with induction (weekly instillations for 6 weeks) followed by 3-year maintenance (weekly instillations for three weeks at 3, 6, 12, 18, 24, 30, and 36 months) (**LE 1; Strong recommendation**).

(4) NICE guideline [5]: Offer induction and maintenance intravesical BCG to people having treatment with intravesical BCG.

(5) Guidelines for Diagnosis and Treatment of Urology and Andrology in China [4]: BCG intravesical instillation starts with induction once a week for a total of 6 times, and maintenance therapy is needed to achieve the best efficacy. There are many maintenance schedules; however, there is no evidence to show that any one is superior to others.

Question 17: For patients with NMIBC, is BCG maintenance instillation for 3 years better than maintenance instillation for 1 year?

(1) EAU guideline [1]: Three-year maintenance is more effective than one year to prevent recurrence in patients with high-risk tumors, but not in patients with intermediate-risk tumors (**LE: 1a**). In patients with intermediate-risk tumors, one-year full-dose BCG treatment (induction plus 3-weekly instillations at 3, 6 and 12 months), or instillations of chemotherapy for a maximum of 1 year is recommended (**Recommendation strength rating: strong**). In patients with high-risk tumors, full-dose intravesical BCG for 1 – 3 years (induction plus 3-weekly instillations at 3, 6, 12, 18, 24, 30 and 36 months), is indicated. The additional beneficial effect of the second and third years of maintenance should be weighed against its added costs, side-effects and problems related to BCG shortage (**Recommendation strength rating: Strong**).

(2) AUA guideline [2]: In intermediate-risk patients who completely respond to induction BCG, maintenance BCG for 1 year, as tolerated, should be considered (Moderate recommendation; Evidence strength: Grade C). In high-risk patients who completely respond to induction BCG, maintenance BCG continued for 3 years, as tolerated, should be administered (**Moderate recommendation; Evidence strength: Grade B**).

(3) CUA guideline [3]: When BCG is administered for intermediate-risk NMIBC, induction (weekly instillations for 6 weeks) followed by 1-year maintenance (weekly instillations for three weeks at 3, 6, and 12 months) is recommended (**LE 1; Strong recommendation**). In patients with high-risk NMIBC, BCG therapy with induction (weekly instillations for 6 weeks) followed by 3-year maintenance (weekly instillations for three weeks at 3, 6, 12, 18, 24, 30, and 36 months) should be administered (**Strong**

recommendation; Evidence strength: Grade B).

(4) NCCN guideline [6]: Although there is no standard regimen for maintenance BCG, many NCCN Member Institutions follow the SWOG regimen consisting of a 6-week induction course of BCG followed by maintenance with 3 weekly instillations at months 3, 6, 12, 18, 24, 30, and 36. Ideally, maintenance should be given for 1 year for intermediate-risk and 3 years for high-risk NMIBC.

Question 18: What is the treatment option after the treatment failure of intravesical BCG immunotherapy?

(1) EAU guideline [1]: Categories of reasons for BCG failure are shown in Table S2. RC is recommended for BCG-unresponsive patients (**Recommendation strength rating: Strong**) (Table S3).

(2) AUA guideline [2]: 1) In an intermediate- or high-risk patient with persistent or recurrent Ta or CIS after a single course of induction intravesical BCG, a second course of BCG should be given (**Moderate recommendation; Evidence strength: Grade C**); 2) In a patient fit for surgery with high-grade T1 disease after a single course of induction intravesical BCG, radical cystectomy should be performed (**Moderate recommendation; Evidence strength: Grade C**); 3) Additional BCG should not be prescribed to a patient who is intolerant of BCG or has documented recurrence on TURBT of high-grade, non-muscle-invasive disease and/or CIS within 6 months of two induction courses of BCG or induction BCG plus maintenance (**Moderate recommendation; Evidence strength: Grade C**); 4) In a patient with persistent or recurrent intermediate- or high-risk NMIBC within 12 months of completion of adequate BCG therapy (two induction courses or one induction course plus one maintenance cycle) who is unwilling or unfit for cystectomy, clinical trial enrollment or alternative intravesical therapy (e.g., valrubicin, gemcitabine, docetaxel, combination chemotherapy) when clinical trials are unavailable is suggested. Systemic immunotherapy with pembrolizumab to a patient with CIS within 12 months of completion of adequate BCG therapy is also recommended (Expert Opinion).

(3) CUA guideline [3]: BCG failure includes: 1) BCG-unresponsive including HG T1 at the first evaluation following induction BCG (at 3 months), recurrent HG Ta/CIS within 6 months of completion of adequate BCG treatment, or recurrent CIS within 12 months of completing adequate BCG treatment; 2) BCG-refractory including any HG T1 after one induction course at 3-month follow up or any HG Ta/CIS after BCG treatment at 6-month follow up; 3) BCG-relapsing disease is defined based on achieving a complete

response to BCG treatment at 6 months but then experiencing any HG recurrence during follow up thereafter; 4) BCG-intolerant which is defined as patients those who experience recurrences after an inadequate course of BCG due to severe adverse effects. 5) RC with pelvic lymph node dissection is the standard of care for BCG-unresponsive bladder cancer in surgically fit patients (**LE 3; Strong recommendation**). 6) For patients with BCG-unresponsive/CIS or HG Ta, a second-line intravesical therapy might be considered before RC (**LE 3; Weak recommendation**). 4) For patients with BCG-unresponsive CIS who are unfit for or refuse to undergo RC, intravenous pembrolizumab, intravesical oportuzumabmonatox, nadofaragenefiradenovec, and BCG plus N-803 have promising efficacy (**LE 2; Weak recommendation**). 8) Alternative options, such as sequential intravesical gemcitabine/docetaxel (induction plus maintenance) may be considered for patients with BCG-unresponsive disease who are unfit for or refuse to undergo RC (LE 3, Weak recommendation). Additional alternatives may also include other combination intravesical therapy (e.g., sequential gemcitabine/MMC, BCG + interferon if available) or single-agent intravesical therapy (MMC, epirubicin, docetaxel, gemcitabine) (**LE 3; Weak recommendation**). 9) Clinical trials may be considered for BCG-unresponsive patients who are unfit for or refuse to undergo RC.

(4) NICE guideline [5]: For people in whom induction BCG has failed, the suitability of RC should be assessed, or further intravesical therapy should be administered if RC is unsuitable or declined by the person, or if the bladder cancer that recurs is intermediate- or low-risk.

(5) Guidelines for Diagnosis and Treatment of Urology and Andrology in China [4]: BCG failure includes: 1) BCG-refractory defined as detection of HG tumors within 6 months of completion of adequate BCG treatment or any grade/stage progression within 3 months after one induction course; 2) BCG-relapsing disease defined as experiencing any HG recurrence (6 – 9 months after the last BCG) after achieving a complete response to BCG treatment at 6 months follow up; 3) BCG-unresponsive including BCG-refractory and BCG-relapsing; 4) RC is recommended for BCG-unresponsive bladder cancer, and for those who are unfit for RC can be offered combination intravesical therapy, or repeat BCG treatment according to patient's situation. For patients with intermediate-risk NMIBC, if non-high-grade recurrence occurs after the BCG treatment, a repeated BCG or RC is feasible.

Table S2 Categories of reasons for BCG failure in EAU guideline

**Category**	**Specific conditions**

Detection of MIBC	Whenever a MIBC is detected during follow-up
BCG-refractory tumor	(1) if T1G3/HG tumor is present at 3 months (LE: 3)
	(2) if TaG3/HG tumor is present after 3 months and/or at 6 months, after either re-induction or first course of maintenance (LE: 4)
	(3) if CIS (without concomitant papillary tumor) is present at 3 months and persists at 6 months after either re-induction or first course of maintenance. If patients with CIS present at 3 months, an additional BCG course can achieve a complete response in > 50% of cases (LE: 1b)
	(4) if HG tumor appears during BCG maintenance therapy*
BCG-relapsing tumor	Recurrence of G3/HG (WHO 1973/2004) tumor after completion of BCG maintenance, despite an initial response (LE: 3)
BCG unresponsive tumor	Recurrence of G3/HG (WHO 1973/2004) tumor after completion of BCG maintenance, despite an initial response (LE: 3)
BCG intolerance	Severe side effects that prevent further BCG instillation before completing treatment

*Patients with low-grade recurrence during or after BCG treatment are not considered to be a BCG failure. *BCG* Bacillus Calmette-Guerin, *CIS* carcinoma in situ, *HG* high grade, *LE* level of evidence, *MIBC* muscle-invasive bladder cancer, *WHO* World Health Organization

Table S3 Treatment options for the various categories of BCG failure in EAU guideline

**Category**	**Treatment options**
BCG-unresponsive	RC
	Enrollment in clinical trials assessing new treatment strategies
	Bladder-preserving strategies in patients unsuitable or refusing RC
Late BCG relapsing:	Radical cystectomy or repeat BCG course according to individual situation
T1Ta/HG recurrence	
> 6 months or CIS	Bladder-preserving strategies

> 12 months of last BCG exposure	
LG recurrence after BCG for primary intermediate-risk tumor	Repeat BCG or intravesical chemotherapy<br>Radical cystectomy

BCG Bacillus Calmette-Guerin, *CIS* carcinoma in situ, *HG* high grade, *LG* low grade, *RC* radical cystectomy

Question 19: How should the side effects of intravesical BCG immunotherapy be managed?

(1) EAU guideline [1]: as shown in Table S4.

(2) CUA guideline [3]: as shown in Table S5.

(3) Guidelines for Diagnosis and Treatment of Urology and Andrology in China [4]: BCG adverse effects are mainly local side effects, manifested as urocystitis, hematuria, fever, reactive arthritis. Patient's symptoms are relieved by suspension of BCG instillation, anti-infection and symptomatic treatment.

Table S4 Management options for side effects associated with intravesical BCG in EAU guideline

**Local side effects**	**Management options (modified from International Bladder Cancer Group)**
Symptoms of cystitis	Phenazopyridine, propantheline bromide, or NSAIDs<br>If symptoms improve within a few days: continue instillations<br>If symptoms persist or worsen: 1) postpone the instillation; 2) perform a urine culture; 3) start empirical antibiotic treatment.<br>If symptoms persist even with antibiotic treatment: 1) with positive culture: adjust antibiotic treatment according to sensitivity; 2) with negative culture: quinolones and potentially analgesic anti-inflammatory instillations once daily for 5 d (repeat cycle if necessary)<br>If symptoms persist: anti-tuberculosis drugs + corticosteroids<br>If no response to treatment and/or contracted bladder: radical cystectomy

Haematuria	Perform urine culture to exclude haemorrhagic cystitis, if other symptoms present<br>If haematuria persists, perform cystoscopy to evaluate presence of bladder tumor
Symptomatic granulomatous prostatitis	Symptoms rarely present: perform urine culture<br>Quinolones<br>If quinolones are not effective: isoniazid (300 mg/d) and rifampicin (600 mg/d) for 3 months<br>Cessation of intravesical therapy
Epididymo-orchitis	Perform urine culture and administer quinolones<br>Cessation of intravesical therapy<br>Orchidectomy if abscess or no response to treatment
**Systemic side effects**	**Management options**
General malaise, fever	Generally, resolve within 48 hours, with or without antipyretics
Arthralgia and/or arthritis	Rare complication and considered autoimmune reaction<br>Arthralgia: treatment with NSAIDs<br>Arthritis: NSAIDs<br>If no/partial response, proceed to corticosteroids, high-dose quinolones or antituberculosis drugs
Persistent high-grade fever<br>(> 38.5°C for > 48 h)	Permanent discontinuation of BCG instillations<br>Immediate evaluation: urine culture, blood tests, chest X-ray<br>Prompt treatment with more than two antimicrobial agents while diagnostic evaluation is conducted<br>Consultation with an infectious diseases specialist
BCG sepsis	Prevention: initiate BCG at least 2 weeks post-transurethral resection of the bladder (if no signs and symptoms of haematuria).

	Cessation of BCG<br>For severe infection: 1) high-dose quinolones or isoniazid, rifampicin and ethambutol 1.2 g daily for 6 months. 2) early, high-dose corticosteroids as long as symptoms persist. 3) consider an empirical non-specific antibiotic to cover Gram-negative bacteria and/or Enterococcus
Allergic reactions	Antihistamines and anti-inflammatory agents<br>Consider high-dose quinolones or isoniazid and rifampicin for persistent symptoms<br>Delay therapy until reactions resolve

BCG Bacillus Calmette-Guérin, *NSAIDs* non-steroidal anti-inflammatory drugs

Table S5 BCG adverse events and management in CUA guideline

**Local side effects**	**Management**
Visible hematuria	Suspend BCG until resolved; laboratory workup (urine, blood, cultures); culture-directed antibiotics for urinary tract infection, if present; if hematuria persists, then perform cystoscopy to rule out bladder cancer recurrence
Epididymitis/prostatitis	Suspend BCG; laboratory workup (urine, blood, cultures); add antibiotics (e.g., quinolones); consider INZ 300 mg/d or RFP 600 mg/d; consider infectious disease consultation; consider orchiectomy
**Systemic side effects**	**Management**
Malaise/ nausea (usually < 48 h)	Symptomatic treatment (e.g., antiemetics).
Allergic reactions	Mild and < 48 h: Antihistamines; NSAID; delay BCG until resolved<br>Persistent: Suspend BCG and consider discontinuing treatment; consider INZ 300 mg/d or RFP 600 mg/d
Fever	< 38.5 ºC and/or < 48 h: Symptomatic treatment (e.g., antipyretics)

	≥ 38.5 ºC for ≥ 48 h: Suspend BCG until resolved/ consider dose reduction; laboratory workup (urine, blood, cultures); start with at least 2 empiric antimicrobials (e.g., quinolones, INZ 300 mg/d, RFP 600 mg/d); consider infectious disease consultation
BCG sepsis	Suspend BCG definitively; hospitalization; laboratory workup (urine, blood, cultures); start empiric antibiotics (e.g., high-dose quinolones); initiate INZ 300 mg/d + RFP 600 mg/d + ethambutol 1200 mg/d, for 6 months); high-dose corticosteroids if persistent (e.g., prednisolone 40 mg/d); infectious disease consultation

BCG Bacillus Calmette-Guérin, *CUA* Canadian Association of Urology, *INZ* isoniazide, *RFP* rifampicin, *NSAIDs* non-steroidal anti-inflammatory drugs, *UTI* urinary tract infection

Question 20: For patients with NMIBC, is combination therapy (intravesical BCG immunotherapy combined with intravesical chemotherapy) better than intravesical BCG immunotherapy alone?

(1) EAU guideline [1]: In one RCT, a combination of mitomycin-C and BCG was shown to be more effective in reducing recurrences but more toxic compared to BCG monotherapy (**LE: 1b**). A RCT showed that a combination of BCG and mitomycin-C with electromotive drug administration improved the recurrence free interval and reduced progression rate compared to BCG monotherapy (**LE: 2**). A RCT comparing BCG monotherapy with a combination of epirubicin and interferon showed the latter was significantly inferior to BCG monotherapy in preventing recurrence (**LE: 1b**).

(2) CUA guideline [3]: Alternative options, such as sequential intravesical gemcitabine/docetaxel (induction plus maintenance) may be considered (**LE 3; Weak recommendation**). Additional alternatives may also include other combination intravesical therapy (e.g., sequential gemcitabine/MMC, BCG+interferon if available) (**LE 3; Weak recommendation**).

Question 21: Is intravesical BCG immunotherapy better than intravesical chemotherapy in patients with CIS?

(1) EAU guideline [1]: Compared to intravesical chemotherapy, BCG treatment of CIS increases the complete response rate, the overall percentage of patients who remain disease free, and reduces the risk of tumor progression. (**LE: 1b**)

(2) AUA guideline [2]: In a high-risk patient with newly diagnosed CIS, high-risk urothelial carcinoma, a 6-week induction course of BCG should be administered. (Strong recommendation; Evidence strength: Grade B). In a high-risk patient who completely responds to induction BCG, maintenance BCG for 3 years should be continued, as tolerated. (**Moderate recommendation; Evidence strength: Grade B**)

(3) CUA guideline [3]: In patients with high-risk NMIBC, BCG therapy with induction (weekly instillations for 6 weeks) followed by 3-year maintenance (weekly instillations for three weeks at 3, 6, 12, 18, 24, 30, and 36 months) is the standard of care for reducing disease recurrence and progression rates. (**LE: 1; Strong recommendation**)

(4) NICE guideline [5]: If the first TURBT shows high-risk NMIBC, offer another TURBT as soon as possible and no later than 6 weeks after the first resection. Offer the choice of intravesical BCG or RC to people with high-risk NMIBC, and base the choice on a full discussion with the patient.

(5) NCCN guideline [6]: Perform post-operative BCG immunotherapy. If the patient is unable to tolerate BCG, intravesical chemotherapy may be considered, but data supporting this approach are limited.

(6) Guidelines for Diagnosis and Treatment of Urology and Andrology in China [4]: Post-operative BCG instillation is recommended for CIS.

Question 22: What are the indications for RC in NMIBC patients?

(1) EAU guideline [1]: Offer a RC to patients with BCG unresponsive tumors (**Recommendation strength rating: Strong**). In patients with high- or very high-risk tumors discuss immediate RC (**Recommendation strength rating: Strong**). RC is feasible for patients with BCG failure. Treatment options for the various categories of BCG failure are shown in Table S3.

(2) AUA guideline [2]: 1)In a patient with Ta low- or intermediate-risk disease, RC should not be performed until bladder-sparing modalities (staged TURBT, intravesical therapies) have failed (Clinical Principle). 2) In a high-risk patient with persistent HG T1 disease on repeat resection, or T1 tumors with associated CIS, LVI, or variant histology, offering initial radical cystectomy should be considered (**Moderate recommendation; Evidence strength: Grade C**). 3) In a high-risk patient with persistent or recurrent disease within one year following treatment with two induction cycles of BCG or BCG maintenance, RC should be offered (**Moderate recommendation; Evidence strength: Grade C**).

(3) CUA guideline [3]: 1) RC with pelvic lymph node dissection is the standard of care for BCG-unresponsive bladder cancer in surgically fit patients (**LE 3; Strong**

recommendation); 2) RC should be considered for patients with large-volume, diffuse, endoscopically unresectable NMIBC (**LE: 3; Strong recommendation**); 3) RC should be offered to patients with HG T1 disease with additional adverse tumor pathological features, including: variant histology (e.g., micropapillary, plasmacytoid, sarcomatoid), extensive invasion of the lamina propria or invasion into or beyond the muscularis mucosa (T1b/c), presence of LVI, concomitant CIS in the bladder or prostatic urethra, multiple and large (≥ 3 cm) tumors, or persistent HG T1 upon re-staging TURBT (**LE3; Strong recommendation**).

(4) NCCN guideline [6]: RC is generally reserved for residual HG cT1, variant histology, lymphovascular invasion, concomitant CIS, and BCG-unresponsive tumors.

(5) Guidelines for Diagnosis and Treatment of Urology and Andrology in China [4]: Patients with BCG-unresponsive tumors should be offered RC. For patients with intermediate-risk NMIBC, if non-high-grade recurrence occurs after the BCG treatment, RC is feasible.

Question 23: Is intravesical BCG immunotherapy superior to intravesical chemotherapy in patients with recurrent NMIBC?

(1) EAU guideline [1]: 1) In patients with tumors presumed to be at low risk and in those with small papillary recurrences detected more than one year after previous TURBT, offer one immediate chemotherapy instillation; 2) For patients with BCG unresponsive tumors, who are not candidates for RC, offer preservation strategies (eg., intravesical chemotherapy, chemotherapy and microwave-induced hyperthermia, electromotive administration of chemotherapy, intravesical- or systemic immunotherapy; preferably within clinical trials) (**Recommendation strength rating: Weak**); 3) Treatment options for the various categories of BCG failure are shown in Table S3.

(2) AUA guideline [2]: 1) In an intermediate- or high-risk patient with persistent or recurrent Ta or CIS disease after a single course of induction intravesical BCG, a second course of BCG should be administered (**Moderate recommendation; Evidence strength: Grade C**). 2) Additional BCG should not be prescribed to a patient who is intolerant of BCG or has documented recurrence on TURBT of HG, non-muscle-invasive disease and/or CIS within 6 months of two induction courses of BCG or induction BCG plus maintenance. (**Moderate recommendation; Evidence strength: Grade C**)

(3) CUA guideline [3]: Patients who develop recurrence during intravesical chemotherapy may be offered induction followed by maintenance BCG (**LE 3; Weak recommendation**). For patients with BCG-unresponsive CIS or HG Ta, a second-line intravesical therapy might be considered before radical cystectomy (**LE 3; Weak recommendation**).

(4) Guidelines for Diagnosis and Treatment of Urology and Andrology in China [4]: Patients with BCG-unresponsive tumors should be offered RC. A patient who is unsuitable

for RC should be treated with bladder-preserving strategies or repeat BCG course according to individual situation. For patients with intermediate-risk NMIBC, if non-high-grade recurrence occurs after the BCG treatment, repeat BCG therapy or radical cystectomy feasible.

Question 24: How to perform follow-up for NMIBC patients after TURBT? (Table S6)

Table S6 Summary of follow-up scheme for NMIBC patients in different guidelines

Follow-up content	Risk level	Guideline	Time after TURBT						
			**1-year**	**2-years**	**3-years**	**4-years**	**5-years**	**6-10 years**	**> 10 years**
Cystoscopy and urine cytology	Low risk	NCCN	Cystoscopy in the 3rd and 12th month (RL: 2a)	Cystoscopy once a year (RL: 2a)				Based on the patient's clinical manifestations (RL: 2a)	
		NICE	Cystoscopy in the 3rd and 12th month	Do not use urinary biomarkers or cytology in addition to cystoscopy. Do not offer routine urinary cytology or prolonged cystoscopy follow-up after 12 months					
		EAU	Cystoscopy in the 3rd and 12th month (RL: weak)	Cystoscopy once a year (RL: weak)				NA	
		CUA	Cystoscopy in the 3rd (RL: weak) and 12th month (RL: weak)	Cystoscopy once a year (RL: weak)					
		AUA	First surveillance cystoscopy within 3 – 4 months (Expert Opinion).<br>If first surveillance cystoscopy is negative for tumor, subsequent surveillance cystoscopy should be performed 6 – 9 months later, and then annually thereafter. (Moderate					Surveillance after five years in the absence of recurrence should be based on shared-decision making	

**Follow-up content**	**Risk level**	**Guideline**	**Time after TURBT**						
			**1-year**	**2-years**	**3-years**	**4-years**	**5-years**	**6-10 years**	**> 10 years**
			Recommendation; Evidence Strength: Grade C)					between the patient and clinician. (Moderate Recommendation; Evidence Strength: Grade C)	
	Intermediate risk	NCCN	Cystoscopy and cytology in the 3rd, 6th and 12th month (RL: 2a)	Cystoscopy and cytology once every 6 months (RL: 2a)	Cystoscopy and cytology once a year (RL: 2a)			Based on the patient's clinical manifestations (RL: 2a)	
		NICE	Cystoscopy in the 3rd, 9th and 18th month		Cystoscopy once a year			If there is no disease progression or recurrence after 5 years of follow-up, the follow-up can be terminated	
		EAU	An in-between (individualized) follow-up scheme using cystoscopy (RL: weak)						
		CUA	Cystoscopy in the 3rd month (recommendation level: strong); then cystoscopy and urine cytology every 3 – 6 months (RL: weak)		Cystoscopy and cytology every 6 – 12 months	Cystoscopy and urine cytology once a year (RL: weak)			

Follow-up content	Risk level	Guideline	Time after TURBT 1-year	2-years	3-years	4-years	5-years	6-10 years	> 10 years
					(RL: weak)				
		AUA	First surveillance cystoscopy within 3 – 4 months (Expert Opinion). If first surveillance cystoscopy is negative for tumor, subsequent surveillance cystoscopy with cytology should be performed 3 – 6 months later (Expert Opinion)		Subsequent surveillance cystoscopy with cytology should be performed 6 – 12 months (Expert Opinion)		Subsequent surveillance cystoscopy with cytology should be performed once a year (Expert Opinion)		
	High risk	NCCN	Cystoscopy and cytology every 3 months (RL: 2a)		Cystoscopy and cytology every 6 months (RL: 2a)			Cystoscopy and cytology once a year (RL: 2a)	Based on the patient's clinical manifestations (RL: 2a)
		NICE	Cystoscopy every 3 months		Cystoscopy every 6 months		Cystoscopy once a year		
		EAU	Cystoscopy and cytology every 3 months (RL: weak)		Cystoscopy and cytology every 6 months (RL: weak)			Cystoscopy and cytology once a year (RL: weak)	
		CUA	Cystoscopy and cytology every 3 – 4		Cystoscopy and cytology		Cystoscopy and urine cytology once a year (RL:		

Follow-up content	Risk level	Guideline	Time after TURBT						
			1-year	2-years	3-years	4-years	5-years	6-10 years	> 10 years
			months (RL: weak)		every 6 months (RL: weak)		weak)		
		AUA	First surveillance cystoscopy within 3 – 4 months (Expert Opinion)<br>If first surveillance cystoscopy is negative for tumor, subsequent surveillance cystoscopy with cytology should be performed 3 – 6 months later (Expert Opinion)		Subsequent surveillance cystoscopy with cytology should be performed for 6 months (Expert Opinion)		Subsequent surveillance cystoscopy with cytology should be performed once a year (Expert Opinion)		
Imaging examination	Low risk	NCCN	Baseline upper urography, abdominal and pelvic imaging (RL: 2a)	Based on the patient's clinical manifestations (RL: 2a)					
		NICE	NA					NA	
		EAU	NA					NA	
		CUA	NA						
		AUA	NA						
	Intermediate risk	NCCN	Baseline upper urography, abdominal and pelvic imaging (RL: 2a)	Based on the patient's clinical manifestations (RL: 2a)					

Follow-up content	Risk level	Guideline	Time after TURBT						
			1-year	2-years	3-years	4-years	5-years	6-10 years	> 10 years
		NICE	NA					NA	
		EAU	NA					NA	
		CUA	NA						
		AUA	Upper tract imaging at 1 – 2 year intervals (Expert Opinion)						
	High risk	NCCN	Upper urinary tract imaging at baseline and 12 months after TURBT, abdominal and pelvic imaging at baseline (RL: 2a)	Upper urinary tract imaging every 1 – 2 years (RL: 2a); Abdominal and pelvic imaging examination is performed according to the patient's clinical manifestations (RL: 2a)					Based on the patient's clinical manifestations (RL: 2a)
		NICE	For patients undergoing radical cystectomy, CT examination of chest, abdomen and pelvis is performed at 6, 12 and 24 months						
		EAU	Regular (yearly) upper tract imaging (CT-IVU or IVU) (RL: weak)						
		CUA	Upper tract imaging in the first year and every 2 years thereafter (RL: weak)						
		AUA	Upper tract imaging at 1 – 2 year intervals (Expert Opinion)						

NMIBC non-muscle invasive bladder cancer, *TURBT* transurethral resection of bladder tumor, *AUA* American Urological Association, *CT* computed tomography, *CUA* Canadian Association of Urology, *EAU* European Association of Urology, *IVU* intravenous urography, *NCCN* National Comprehensive Cancer Network, *NICE* The National Institute for Health and Care Excellence, *NA* not applicable, *RL* recommendation level

References

1. Eau. EAU-ESTRO-SIOG Guidelines on Non-muscle-invasive Bladder Cancer. 2021. https://uroweb.org/guideline/non-muscle-invasive-bladder-cancer/.
2. Chang S, Bochner B, Chou R, Clark P. Diagnosis and Treatment of Non-Muscle Invasive Bladder Cancer: AUA/SUO Joint Guideline (2020). 2021. https://www.auanet.org/guidelines/guidelines/bladder-cancer-non-muscle-invasive-guideline.
3. Bimal B, Ronald K, Girish S, Siemens D, Armen G. Canadian Urological Association guideline on the management of non-muscle invasive bladder cancer 2021. https://www.cua.org/system/files/Guidelines/7367_NMIBC%20Guideline_Epub.pdf.
4. Guo YL, Na YQ, Ye ZQ, Huang J. Guidelines for Diagnosis and Treatment of Urology and Andrology in China. Beijing: Science Press; 2019.
5. National Institute for Health and Care Excellence (Nice). Bladder cancer: diagnosis and management. 2019. https://www.nice.org.uk/guidance/ng2.
6. National Comprehensive Cancer Network (Nccn). NCCN clinical practice guidelines in oncology: Bladder cancer. 2021. https://www.nccn.org/guidelines/guidelines-detail?category=1&id=1417.
7. Urology Branch of Chinese Medical Association, China Bladder Cancer Federation. Chinese expert consensus on secondary resection of non-muscle-invasive bladder cancer. Chin J Urol. 2017;38(8):561-3.
