# Supplementary material for: Treatment and surveillance for non-muscle-invasive bladder cancer: a clinical practice guideline (2021 edition)
Source: Mil Med Res. 2022 Aug 17;9:44. doi: 10.1186/s40779-022-00406-y (PMC9382792; doi:10.1186/s40779-022-00406-y)
Supplement: Supplementary file 3 — Additional file 3. Results of meta-analyses [file 40779_2022_406_MOESM3_ESM.pdf]

Table S1 Meta-analysis results of chemotherapy drug subgroup

Drug	Subgroup		Outcome [*RR* (95%CI)]									
			Recurrence rate					Bladder irritation sign	Hematuria	Cystitis	Fever	Allergy
			1-year	2-year	3-year	4-year	5-year					
Gemcitabine vs. Pirarubicin	Dosage (mg)	1000 vs. 30 – 50	0.51 (0.33, 0.77)*	0.59 (0.48, 0.73)*	0.51 (0.31, 0.84)*	NA	NA	0.52 (0.42, 0.64)*	0.38 (0.24, 0.58)*	0.35 (0.14, 0.86)*	1.28 (0.62, 2.65)	0.42 (0.14, 1.26)
		1000 vs. < 30	0.13 (0.02, 0.95)*	0.46 (0.17, 1.19)	NA	NA	NA	NA	NA	NA	NA	NA
		< 1000 vs. 30 - 50	1.35 (0.57, 3.17)	0.64 (0.29, 1.42)	NA	NA	NA	0.50 (0.15, 1.65)	0.16 (0.04, 0.61)*	NA	0.25 (0.06, 1.10)	0.50 (0.05, 5.23)
		> 1000 vs. 30 – 50	NA	0.39 (0.16, 0.95)*	NA	NA	NA	0.84 (0.41, 1.72)	NA	NA	NA	NA
	Medication scheme	SIC in both groups	NA	NA	0.41 (0.20, 0.83)*	NA	NA	0.67 (0.14, 3.30)	0.08 (0.01, 0.56)*	NA	1.83 (0.76, 4.43)	NA
		SIC + induction + maintenance in both groups	0.47 (0.28, 0.80)*	0.58 (0.46, 0.72)*	0.83 (0.29, 2.41)	NA	NA	0.50 (0.38, 0.67)*	0.41 (0.24, 0.67)*	0.35 (0.14, 0.86)*	0.41 (0.15, 1.15)	0.33 (0.10, 1.03)
		Induction only in both groups	1.25 (0.37, 4.26)	NA	NA	NA	NA	0.20 (0.03, 1.62)	0.33 (0.04, 3.05)	NA	NA	NA
		Induction + maintenance in both groups	0.63 (0.31, 1.30)	0.59 (0.41, 0.85)*	0.51 (0.19, 1.37)	NA	NA	0.60 (0.43, 0.83)*	0.29 (0.13, 0.69)*	NA	0.34 (0.01, 8.14)	1.00 (0.07, 15.43)
Pirarubicin vs. Hydroxycamptothecin	Dosage (mg)	30 – 50 vs. > 20	NA	4.00 (0.92, 17.35)	NA	NA	NA	NA	NA	NA	NA	NA
		30 – 50 vs. 10 – 20	0.79 (0.58, 1.07)	0.75 (0.57, 0.99)*	0.84 (0.37, 1.91)	0.92 (0.44, 1.95)	0.73 (0.43, 1.23)	1.39 (1.14, 1.70)*	0.99 (0.60, 1.62)	NA	NA	NA

	Medication scheme	Induction + maintenance in both groups	0.72 (0.46, 1.13)	0.75 (0.51, 1.10)	0.84 (0.37, 1.91)	0.92 (0.44, 1.95)	0.73 (0.43, 1.23)	1.40 (1.09, 1.79)*	1.19 (0.56, 2.53)	NA	NA	NA
		SIC +induction+ maintenance in both groups	0.85 (0.55, 1.32)	0.95 (0.59, 1.53)	NA	NA	NA	1.38 (0.94, 2.02)	0.85 (0.43, 1.65)	NA	NA	NA
Pirarubicin vs. mitomycin-C	Dosage (mg)	30 vs. 20	0.40 (0.23, 0.69)*	0.42 (0.28, 0.63)*	0.57 (0.23, 1.45)§	NA	0.16 (0.02, 1.29)	0.62 (0.32, 1.20)	0.32 (0.09, 1.13)	0.94 (0.37, 2.39) §	NA	NA
		30 vs. 30	0.33 (0.11, 0.98)*	0.54 (0.33, 0.90)*	NA	NA	NA	0.46 (0.28, 0.75)*	0.99 (0.14, 7.23) §	NA	NA	NA
		30 vs. 40	0.66 (0.42, 1.03)	0.35 (0.21, 0.57)*	NA	NA	NA	0.76 (0.51, 1.12)	0.59 (0.28, 1.24)	NA	NA	NA
		40 vs. 40	0.31 (0.09, 1.11)	0.53 (0.34, 0.82)*	0.64 (0.31, 1.34)	NA	NA	0.58 (0.35, 0.94)*	0.20 (0.02, 1.65)	NA	NA	NA
	Medication scheme	Induction only in both groups	0.25 (0.03, 2.11)	NA	NA	NA	NA	NA	0.33 (0.04, 3.03)	NA	NA	NA
		Induction + maintenance in both groups	0.54 (0.37, 0.80)*	0.44 (0.33, 0.57)*	0.57 (0.23, 1.45) §	NA	NA	0.61 (0.43, 0.87)*	0.55 (0.27, 1.12)	NA	NA	NA
		SIC + induction + maintenance in both groups	0.45 (0.26, 0.77)*	0.50 (0.33, 0.75)*	0.64 (0.31, 1.34)	NA	0.16 (0.02, 1.29)	0.59 (0.44, 0.80)*	0.50 (0.16, 1.58) §	NA	NA	NA

*There was significant difference between the two groups; §there is heterogeneity among the studies included in this outcome indicator. *CI* confidence interval, *RR* relative risk, *SIC* single postoperative instillation of intravesical chemotherapy

Table S2 Subgroup analysis results of intravesical BCG immunotherapy

**Subgroup**		**Outcome [RR (95%CI)]**														
		**Recurrence rate**					**Progress rate**					**Bladder irritation sign**	**Hematuria**	**Cystitis**	**Fever**	**Allergy**
		**1-year**	**2-year**	**3-year**	**4-year**	**5-year**	**1-year**	**2-year**	**3-year**	**4-year**	**5-year**					
BCG instillation time	≤ 1 year	1.22 (0.64, 2.32)	0.48 (0.30, 0.79)*	0.52 (0.28, 0.97)*	NA	1.06 (0.70, 1.62) §	0.69 (0.20, 2.37)	0.67 (0.21, 2.18)	0.28 (0.06, 1.37)	0.61 (0.33, 1.12)	0.84 (0.50, 1.39)	4.69 (2.37, 9.31)*	2.61 (1.75, 3.90)*	2.81 (1.96, 4.04)* §	8.53 (3.76, 19.37)*	0.44 (0.17, 1.13)
	> 1 year	1.32 (0.95, 1.85)	0.65 (0.36, 1.19) §	0.94 (0.60, 1.48)	1.84 (0.73, 4.68)	0.47 (0.34, 0.63)*	NA	0.25 (0.02, 3.87)	NA	0.54 (0.27, 1.07)	0.78 (0.54, 1.12)	2.12 (1.41, 3.19)* §	1.54 (1.30, 1.84)*	1.57 (1.01, 2.42)* §	3.83 (2.06, 7.14)*	3.40 (0.14, 81.76)
BCG dose	< 80 mg	0.76 (0.43, 1.37)	NA	NA	NA	0.74 (0.60, 0.92)*	0.69 (0.20, 2.37)	NA	NA	0.61 (0.33, 1.12)	0.84 (0.50, 1.39)	NA	NA	0.23 (0.01, 4.62)	NA	3.40 (0.14, 81.76)
	80 – 120 mg	1.49 (1.19, 1.88)*	0.60 (0.38, 0.96)* §	0.97 (0.66, 1.44)	NA	0.35 (0.18, 0.69)*	NA	0.58 (0.20, 1.70)	NA	0.88 (0.19, 4.02)	NA	2.41 (1.36, 4.25)* §	2.05 (1.49, 2.81)*	3.28 (2.15, 5.02)* §	4.12 (2.37, 7.25)*	NA
	> 120 mg	NA	NA	0.34 (0.21, 0.56)*	1.84 (0.73, 4.68)	NA	NA	NA	0.28 (0.06, 1.37)	NA	NA	3.67 (0.31, 42.80) §	1.47 (1.07, 2.02)*	2.35 (1.65, 3.34)*	5.60 (3.21, 9.75)*	NA
BCG instillation scheme	Induction only	NA	NA	NA	NA	1.29 (0.93, 1.77)	NA	NA	NA	NA	NA	NA	NA	1.57 (1.30, 1.88)*	NA	0.44 (0.17, 1.13)
	Induction + maintenance	1.33 (1.05, 1.69)*	0.60 (0.38, 0.97)*	0.69 (0.44, 1.07)	1.84 (0.73, 4.68)	0.56 (0.40, 0.78)*	0.69 (0.20, 2.37)	0.58 (0.20, 1.70)	0.28 (0.06, 1.37)	0.58 (0.36, 0.91)*	0.80 (0.59, 1.07)	2.41 (1.57, 3.69)*	1.80 (1.46, 2.21)*	2.71 (1.78, 4.12)* §	4.70 (3.09, 7.14)*	3.40 (0.14, 81.76)

*There was significant difference between the two groups; §there is heterogeneity among the studies included in this outcome indicator. *BCG* Bacillus Calmette-Guérin, *CI* confidence interval, *RR* relative risk, *NA* not available

Table S3 Subgroup analysis results of low dose and standard dose of BCG

**Indicator**	**Instillation scheme**	**Studies included**	**Heterogeneity**		**HR（95%CI）**
			I^2 **(%)**	***P***	
Recurrence free risk	Simple induction	5	0.0	0.812	1.40 (0.92, 2.15)
	Induction plus maintenance	4	0.0	0.942	1.20 (1.04, 1.37)
Progression free risk	Simple induction	3	0.0	0.876	1.30 (0.51, 3.31)
	Induction plus maintenance	4	0.0	0.985	1.08 (0.84, 1.38)
Total adverse reactions	Simple induction	4	54.4	0.087	0.53 (0.32, 0.91)
	Induction plus maintenance	8	68.4	0.002	0.66 (0.51, 0.87)
Serious adverse reactions	Simple induction	3	0.0	0.954	0.23 (0.04, 1.26)
	Induction plus maintenance	3	0.0	0.867	0.54 (0.37, 0.78)

BCG Bacillus Calmette-Guérin, *CI* confidence interval, *HR* hazard risk

Table S4 Subgroup analysis results of BCG induction plus maintenance instillation and simple induction instillation

Indicators	Subgroup		Studies included	Heterogeneity		*HR*/*RR*（95%CI）
				I^2(%)	*P*	
Recurrence survival risk	Maintenance time	≥ 2 years	3	14.5	0.310	0.64 (0.51, 0.80)*
		< 2 years	7	31.7	0.186	0.58 (0.40, 0.84)*
	Maintenance dose	≤ 81 mg	7	3.7	0.398	0.61 (0.48, 0.76)*
		> 81 mg	3	58.5	0.090	0.61 (0.28, 1.32)*
	Risk level	Intermediate/high-risk	7	38.0	0.139	0.59 (0.48, 0.73)*
		High-risk	3	0.0	0.445	0.35 (0.20, 0.63)*
Progression survival risk	Maintenance time	≥ 2 years	3	0.0	0.999	0.74 (0.57, 0.96)*
		< 2 years	4	0.0	0.741	0.54 (0.26, 1.14)*
	Maintenance dose	≤ 81 mg	5	0.0	0.768	0.71 (0.54, 0.93)*
		> 81 mg	2	0.0	0.985	0.74 (0.38, 1.46)*
	Risk level	Intermediate/high-risk	7	0.0	0.934	0.71 (0.56, 0.92)*
		High-risk	3	0.0	0.712	0.67 (0.28, 1.61)*
Overall rate of recurrence and progression	Maintenance time	≥ 2 years	3	34.2	0.219	0.86 (0.71, 1.05)§
		< 2 years	9	60.9	0.009	0.70 (0.47, 1.04)§
	Maintenance dose	≤ 81 mg	7	48.5	0.100	0.67 (0.47, 0.97)§
		> 81 mg	5	60.8	0.018	0.88 (0.63, 1.22)§
	Risk level	Intermediate/high-risk	9	62.3	0.007	0.72 (0.55, 0.93)§
		High-risk	6	27.8	0.226	0.70 (0.55, 0.89)§

*The index is *HR*; §the index is *RR*. *BCG* Bacillus Calmette-Guérin, *CI* confidence interval, *HR* hazard risk, *RR* relative risk
